# TTBK2 circular RNA promotes glioma malignancy by regulating miR-217/HNF1β/Derlin-1 pathway

**DOI:** 10.1186/s13045-017-0422-2

**Published:** 2017-02-20

**Authors:** Jian Zheng, Xiaobai Liu, Yixue Xue, Wei Gong, Jun Ma, Zhuo Xi, Zhongyou Que, Yunhui Liu

**Affiliations:** 1 Department of Neurosurgery, Shengjing Hospital of China Medical University, Shenyang, 110004 People’s Republic of China; 2 Liaoning Research Center for Translational Medicine in Nervous System Disease, Shenyang, 110004 People’s Republic of China; 3 Department of Neurobiology, College of Basic Medicine, China Medical University, Shenyang, 110122 People’s Republic of China; 4 Key Laboratory of Cell Biology, Ministry of Public Health of China, and Key Laboratory of Medical Cell Biology, Ministry of Education of China, China Medical University, Shenyang, 110122 People’s Republic of China

**Keywords:** Circular RNAs, circ-TTBK2, MicroRNAs, miR-217, Glioma

## Abstract

**Background:**

Circular RNAs are a subgroup of non-coding RNAs and generated by a mammalian genome. Herein, the expression and function of circular RNA circ-TTBK2 were investigated in human glioma cells.

**Methods:**

Fluorescence in situ hybridization and quantitative real-time PCR were conducted to profile the cell distribution and expression of circ-TTBK2 and microRNA-217 (miR-217) in glioma tissues and cells. Immunohistochemical and western blot were used to determine the expression of HNF1β and Derlin-1 in glioma tissues and cells. Stable knockdown of circ-TTBK2 or overexpression of miR-217 glioma cell lines (U87 and U251) were established to explore the function of circ-TTBK2 and miR-217 in glioma cells. Further, luciferase reports and RNA immunoprecipitation were used to investigate the correlation between circ-TTBK2 and miR-217. Cell Counting Kit-8, transwell assays, and flow cytometry were used to investigate circ-TTBK2 and miR-217 function including cell proliferation, migration and invasion, and apoptosis, respectively. ChIP assays were used to ascertain the correlations between HNF1β and Derlin-1.

**Results:**

We found that circ-TTBK2 was upregulated in glioma tissues and cell lines, while linear TTBK2 was not dysregulated in glioma tissues and cells. Enhanced expression of circ-TTBK2 promoted cell proliferation, migration, and invasion, while inhibited apoptosis. MiR-217 was downregulated in glioma tissues and cell lines. We also found that circ-TTBK2, but not linear TTBK2, acted as miR-217 sponge in a sequence-specific manner. In addition, upregulated circ-TTBK2 decreased miR-217 expression and there was a reciprocal negative feedback between them in an Argonaute2-dependent manner. Moreover, reintroduction of miR-217 significantly reversed circ-TTBK2-mediated promotion of glioma progression. HNF1β was a direct target of miR-217, and played oncogenic role in glioma cells. Remarkably, circ-TTBK2 knockdown combined with miR-217 overexpression led to tumor regression in vivo.

**Conclusions:**

These results demonstrated a novel role circ-TTBK2 in the glioma progression.

**Electronic supplementary material:**

The online version of this article (doi:10.1186/s13045-017-0422-2) contains supplementary material, which is available to authorized users.

## Background

Human malignant gliomas are the most common and lethal primary brain tumor in adults [1, 2], and glioma cells are featured as carrying heterogeneous genetic molecular aberrations [3–5]. Despite the application of advanced chemotherapy, radiotherapy, and surgery, patients with this disease suffer a badly median survival [6–8]. Hence, deeper elucidation of the molecular mechanisms underlying glioma malignancy may offer improved treatments of gliomas.

It has been confirmed that a large proportion (>90%) of the human genome is actively transcribed, and most of the transcripts are identified as non-coding RNAs (ncRNAs) [9]. Circular RNAs (circRNAs), referred to as ncRNAs, are different from the linear RNAs with a circular structure by joining the 3′ end of the RNA to the 5′ end [10, 11]. Recently, circRNAs have been found to have multiple functions in various mammalian cells. Most circRNAs are derived from exons, and most of them are located in the cytoplasm [12]. Accumulated evidence showed that circRNAs harbor microRNA (miRNA) binding sites and function as miRNA sponges. For example, miR-138 targets the circular RNA SRY in a sequence-specific manner [13]. In addition, the circular RNA CiRS-7 possess a binding site of miR-7 and modulate miR-7 expression [14]. Tau tubulin kinase 2 (TTBK2) was first identified as a kinase which phosphorylated tau and tubulin [15]. Aberrant expression of linear TTBK2 is related to various diseases. Overexpression of TTBK2 contributes to the progression of amyotrophic lateral sclerosis [16]. More importantly, increased expression of TTBK2 attenuates the sunitinib-induced apoptosis of kidney carcinoma and melanoma cell lines [17]. Besides, our preliminary experiment demonstrated that TTBK2 circular RNA (circ-TTBK2, also named has_circ_0000594 according to circBase) was upregulated in glioma tissues. Therefore, we hypothesized that dysregulation of circ-TTBK2 was involved in the regulation of glioma malignancy.

MiRNAs are characterized as a member of small non-coding RNAs and have been confirmed to be involved in both biological and pathological processes [18]. Expression profiling analysis has depicted a possible tumor-suppressive function of miR-217 in various cancers. As previously reported, miR-217 is robustly downregulated in human epithelial ovarian cancer (EOC) and inhibits cell growth and metastasis [19]. Additionally, miR-217 expression is downregulated and exerts a tumor-suppressive function in gastric cancer [20]. Bioinformatics software (Starbase) revealed that circ-TTBK2 harbor a binding site of miR-217. However, the function of miR-217 in glioma and circ-TTBK2/miR-217 functional network in modulating glioma malignant behavior remains unknown.

Hepatocyte nuclear factors (HNFs) were initially identified as a group of transcription factors that were involved in regulating the transcription of liver-specific genes. Hepatocyte nuclear factor-1beta (HNF1β) is the most important member of liver-specific transcription factor and is responsible for sequence-specific DNA binding. It is a homeobox transcription factor functioning as a homodimer or heterodimer with HNF1α [21]. In addition, HNF1β has been characterized as an oncogene in various tumors. HNF1β is upregulated in hepatocellular carcinoma (HCC), and high level of HNF1β leads to poor overall survival [22]. Also, HNF1β promotes malignant progression of ovarian clear cell carcinoma via facilitating glucose uptake and glycolytic activity [23]. More importantly, using bioinformatics softwares (Targetscan, miRanda, and RNAhybrid), a binding site was identified between miR-217 and HNF1β. However, the potential oncogenic function of HNF1β in glioma remains poorly defined.

Derlin-1 participates in the dislocation of misfolded proteins from endoplasmic reticulum (ER) and protects cancer cells from endoplasmic reticulum stress-induced apoptosis. Moreover, Derlin-1 expression is upregulated in various tumors such as human breast carcinoma and colon tumor [24, 25]. Besides, previous reports unveiled that overexpressed Derlin-1 activated PI3K/AKT and ERK signaling pathways [26, 27]. Also, by scanning the promoter sequence of Derlin-1, we found a putative binding site of HNF1β. Although the oncogenic role of Derlin-1 is confirmed in many tumors, whether Derlin-1 exerts oncogene function in glioma remains unclear.

In the present study, we investigated the expression and functions of circ-TTBK2, miR-217, HNF1β, and Derlin-1 in glioma tissues and cells. Circ-TTBK2, but not linear TTBK2, exerted oncogenic role in glioma cells. Furthermore, miR-217 targeted circ-TTBK2 in a sequence-specific manner, miR-217 and circ-TTBK2 formed a negative feedback loop possibly mediated by RNA-induced silencing complex (RISC). Moreover, HNF1β was confirmed to harbor a binding site of miR-217 using dual-luciferase assays. These results demonstrated a detailed function of circ-TTBK2 in glioma and provided a novel potential approach for glioma therapy.

## Results

### Circ-TTBK2, but not linear TTBK2, was upregulated in glioma tissues and cell lines and played oncogenic function in glioma cells

**Fig. 1:**
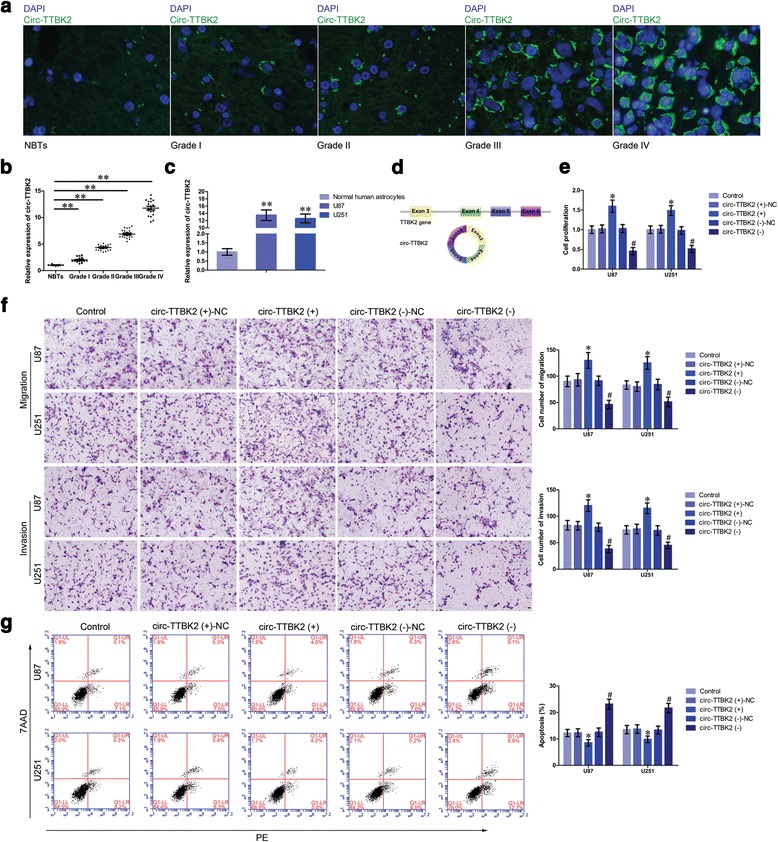
Circ-TTBK2 expression in glioma tissues and cell lines, enforced circ-TTBK2 promoted malignant progression of U87 and U251 glioma cells. **a** FISH was used to determine expression and location of circ-TTBK2 in glioma tissues and normal brain tissues (NBTs) (*green*, circ-TTBK2; *blue*, DAPI nuclear staining). Scale bars represent 20 μm. **b** qRT-PCR was conducted to detect expression levels of circ-TTBK2 in glioma tissues of different grades and NBTs (data are presented as the mean + SD (*n* = 11, NBTs group; *n* = 19, each glioma tissue group). ^**^ *P* < 0.01 vs. NBTs group). **c** Expression levels of circ-TTBK2 in human normal astrocytes and glioma cell lines (data are presented as the mean + SD (*n* = 5, each group). ^**^ *P* < 0.01 vs. normal human astrocyte group). **d** Cartoon of circ-TTBK2 arose from TTBK2 gene. **e** CCK-8 assay was used to determine the proliferation effect of circ-TTBK2 on U87 and U251 cells. **f** Quantification number of migration and invasion cells with overexpression or knockdown of circ-TTBK2. Representative images and accompanying statistical plots were presented. **g** Flow cytometry analysis of U87 and U251 cells with the expression of circ-TTBK2 changed (data are presented as the mean + SD (*n* = 5, each group). ^*^ *P <* 0.05 vs. circ-TTBK2 (+)-NC group; ^*#*^ *P <* 0.05 vs. circ-TTBK2 (−)-NC group. Scale bars represent 40 μm)

By scanning TTBK2 genome and circBase, we found that circ-TTBK2 contained only exons and was generated from exon 3, exon 4, exon 5, and exon 6 from TTBK2 gene (Fig. 1d). The expression of circ-TTBK2 in glioma tissues and cells was determined by fluorescence in situ hybridization (FISH) and quantitative real-time PCR (qRT-PCR). Circ-TTBK2 was located in the cytoplasm and was significantly upregulated in glioma tissues compared with normal brain tissues (Fig. 1a). Circ-TTBK2 expression was significantly increased in glioma tissues and cells (Fig. 1b, c). And the expression of circ-TTBK2 was positively correlated with the pathological grades of glioma. However, linear TTBK2 expression was not changed in glioma tissues and cells (Additional file 1: Figure S1a, b). Furthermore, RNase R was used to confirm the circular form RNA was detected. As expected, circ-TTBK2 was resistant to RNase R treatment, while linear TTBK2 was significantly reduced in cells treated with RNase R (Additional file 1: Figure S1c, d). These results showed that circ-TTBK2 dysregulation might contribute to the malignant progression of glioma cells. Stable circ-TTBK2 overexpression and inhibition in glioma cells were established to investigate the role of circ-TTBK2. To confirm the circular form of TTBK2 was force-expressed instead of the linear form, we examined transfection efficiency and the TTBK2 expression after transfection of circ-TTBK2. As expected, circ-TTBK2 expression was significantly higher in the circ-TTBK2 (+) group, while its expression was significantly lower in the circ-TTBK2 (−) group (Additional file 1: Figure S1e). Also, no significant change of TTBK2 neither in the circ-TTBK2 (+) group nor circ-TTBK2 (−) group (Additional file 2: Figure S2a). We also examined circ-TTBK2 expression in cells treated with sh-TTBK2 (transfection efficiency of sh-TTBK2 data was shown in Additional file 1: Figure S1f). The results showed that sh-TTBK2 did not influence circ-TTBK2 expression (Additional file 2: Figure S2b). Inhibition of circ-TTBK2 led to a significant decrease in the proliferation compared with the circ-TTBK2 (−)-NC group (Fig. 1e). Transwell assay was used to measure whether circ-TTBK2 dysregulation could affect the migration and invasion of glioma cells. Cell number in the circ-TTBK2 (+) group was obviously increased compared with that in the circ-TTBK2 (+)-NC group (Fig. 1f). Moreover, overexpressed circ-TTBK2 hindered apoptosis of glioma cells compared with the circ-TTBK2 (+)-NC group (Fig. 1g).

### MiR-217 was downregulated in glioma tissues and cell lines and exerted tumor-suppressive function in glioma cells

**Fig. 2:**
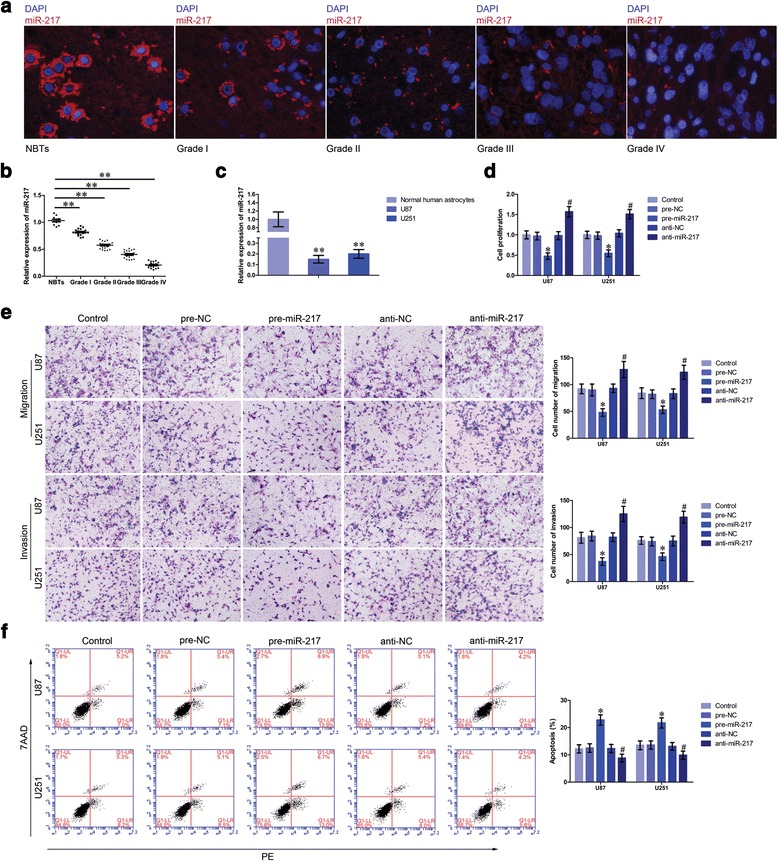
miR-217 expression in glioma tissues and cell lines: restoration of miR-217 restrained the malignant progression of glioma cells. **a** FISH was used to determine expression and location of miR-217 in glioma tissues and normal brain tissues (NBTs) (*red*, miR-217; *blue*, DAPI nuclear staining). Scale bars represent 20 μm. **b** qRT-PCR was conducted to detect expression levels of miR-217 in glioma tissues of different grades and NBTs (data are presented as the mean + SD (*n* = 11, NBTs group; *n* = 19, each glioma tissue group). ^**^ *P* < 0.01 vs. NBT group). **c** Expression levels of miR-217 in human normal astrocytes and glioma cell lines (data are presented as the mean + SD (*n* = 5, each group). ^**^ *P* < 0.01 vs. normal human astrocyte group). **d** CCK-8 assay was applied to evaluate the proliferation effect of miR-217 on U87 and U251 cells. **e** Quantification number of migration and invasion cells with different expression levels of miR-217. Representative images and accompanying statistical plots were presented. Scale bars represent 40 μm. **f** Flow cytometry analysis of U87 and U251 cells with the expression of miR-217 changed (data are presented as the mean + SD (*n* = 5, each group). ^*^ *P <* 0.05 vs. pre-NC group; ^*#*^ *P <* 0.05 vs. anti-NC group)

MiR-217 expression levels in glioma tissues and cells were analyzed by FISH and qRT-PCR. MiR-217 expression was negatively correlated with the pathological grades of glioma, and similarly with circ-TTBK2, miR-217 was also located in the cytoplasm (Fig. 2a, b). Meanwhile, the expression of miR-217 was lower in U87 and U251 glioma cells than that in normal human astrocytes (Fig. 2c). After transfection, we first examined the transfection efficiency (Additional file 1: Figure S1g). Restoration of miR-217 significantly inhibited glioma cell proliferation compared with the pre-NC group (Fig. 2d). Also, overexpressed miR-217 decreased migrating and invading cell numbers compared with that in the pre-NC group (Fig. 2e). MiR-217 overexpression promoted apoptosis of glioma cells compared with the pre-NC group (Fig. 2f). This inferred that miR-217 might function as a tumor-suppressor in glioma cells.

### MiR-217 functional targeted circ-TTBK2, but not TTBK2, and reversed the circ-TTBK2-mediated promotion of glioma cell progression

**Fig. 3:**
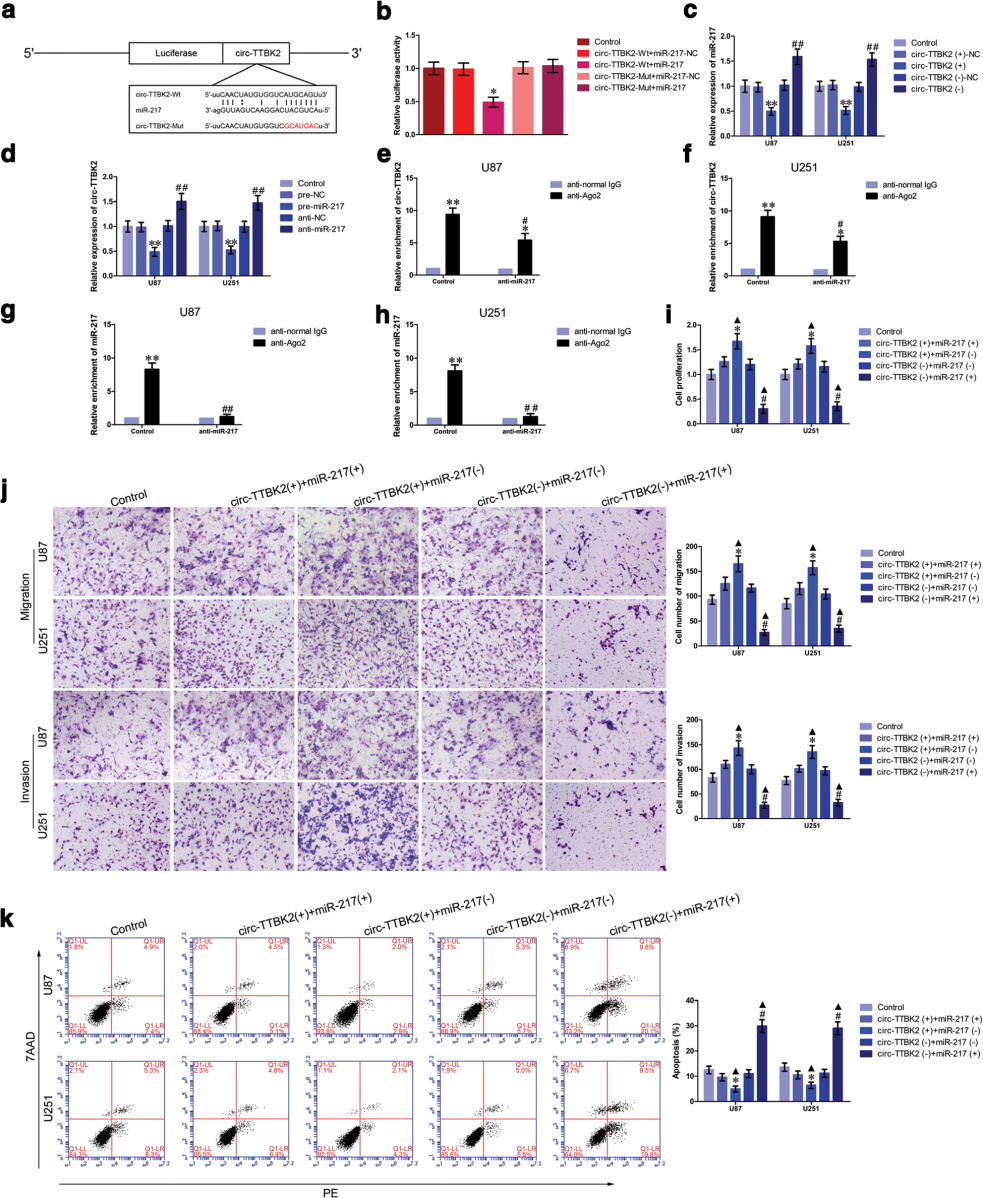
Overexpression of circ-TTBK2 promoted malignant biological behavior of glioma cells via decreasing miR-217 expression. **a** The predicted miR-217 binding site in circ-TTBK2 (circ-TTBK2-Wt) or and the designed mutant sequence (circ-TTBK2-Mut) were indicated. **b** Luciferase reporter assay of HEK 293T cells co-transfected with circ-TTBK2-Wt or circ-TTBK2-Mut and miR-217 or the miR-217-NC (data are presented as the mean + SD (*n* = 5, each group). ^*^ *P* < 0.05 vs. circ-TTBK2-Wt+miR-217-NC group). **c** qRT-PCR analysis for circ-TTBK2 regulated miR-217 expression in U87 and U251 cells (data are presented as the mean + SD (*n* = 5, each group). ^**^ *P* < 0.01 vs. circ-TTBK2 (+) group; ^##^ *P* < 0.01 vs. circ-TTBK2 (−)-NC group). **d** qRT-PCR analysis for miR-217 attenuated circ-TTBK2 expression in U87 and U251 cells (data are presented as the mean + SD (*n* = 5, each group). ^**^ *P* < 0.01 vs. pre-NC group; ^##^ *P* < 0.01 vs. anti-NC group). **e**–**h** miR-217 was identified in circ-TTBK2-RISC complex. Relative enrichment of circ-TTBK2 and miR-217 were measured using qRT-PCR (data represent mean + SD (*n* = 5, each group). ^*^ *P* < 0.05, ^**^ *P* < 0.01 vs. anti-normal IgG respective group, ^#^ *P* < 0.05, ^##^ *P* < 0.01 vs. anti-Ago2 in control group). **i** CCK-8 assay was applied to evaluate the proliferation effect of circ-TTBK2 and miR-217 on U87 and U251 cells (data are presented as the mean + SD (*n* = 5, each group). ^*^ *P <* 0.05 vs. circ-TTBK2 (+)+miR-217 (+) group; ^*#*^ *P <* 0.05 vs. circ-TTBK2 (−)+miR-217(−) group; ^▲^ *P <* 0.05 vs. control group). **j** Quantification number of migration and invasion cells with the expression of circ-TTBK2 and miR-217 changed. Representative images and accompanying statistical plots were presented (data are presented as the mean + SD (*n* = 5, each group). ^*^ *P <* 0.05 vs. circ-TTBK2 (+)+miR-217 (+) group; ^*#*^ *P <* 0.05 vs. circ-TTBK2 (−)+miR-217(−) group; ^▲^ *P <* 0.05 vs. control group. Scale bars represent 40 μm). **k** Flow cytometry analysis of U87 and U251 with the expression of circ-TTBK2 and miR-217 changed (data are presented as the mean + SD (*n* = 5, each group). ^*^ *P <* 0.05 vs. circ-TTBK2 (+)+miR-217 (+) group; ^*#*^ *P <* 0.05 vs. circ-TTBK2 (−)+miR-217(−) group; ^▲^ *P <* 0.05 vs. control group)

As previously mentioned, circRNAs could function as miRNAs sponge, and miRNAs could target circRNAs in a sequence-specific manner. We assumed that circ-TTBK2 might harbor a binding site of miR-217 using bioinformatics database (Starbase). To verify our prediction, dual-luciferase gene reporter assay was conducted. The luciferase activity in the circ-TTBK2-Wt+miR-217 group was significantly decreased than that in the circ-TTBK2-Wt+miR-217-NC group (Fig. 3b), while the luciferase activity in the circ-TTBK2-Mut+miR-217 group was not changed. Two putative binding sites were identified in linear TTBK2. However, the luciferase assays TTBK2-Wt+miR-217 did not influence the luciferase activity, while TTBK2-Wt1+miR-217 decreased the luciferase activity (Additional file 2: Figure S2e, f). Further, we measured miR-217 expression in the circ-TTBK2 (+) and circ-TTBK2 (−) groups by qRT-PCR. MiR-217 expression was significantly decreased in the circ-TTBK2 (+) group, while miR-217 expression was significantly increased in the circ-TTBK2 (-) group (Fig. 3c). Similarly, the expression of circ-TTBK2 was significantly restored in anti-miR-217 group (Fig. 3d). Also, we found that overexpression or downregulation of miR-217 did not affect the expression of TTBK2 (Additional file 2: Figure S2c).

RNA-binding protein immunoprecipitation (RIP) assay was conducted to investigate whether circ-TTBK2 and miR-217 were involved in the expected RNA-induced silencing complex (RISC) complex. RNA levels in immunoprecipitates were detected by qRT-PCR. The relative enrichment levels of circ-TTBK2 and miR-217 were both increased in the anti-Ago2 group than those in the anti-normal group. In the anti-miR-217 group, the relative enrichment levels of circ-TTBK2 and miR-217 immunoprecipitated with Ago2 were lower than those in the control group, respectively (Fig. 3e–h). In summary, these results indicated that circ-TTBK2 reduced miR-217 expression in RISC manner, and there might be a reciprocal repression feedback loop between circ-TTBK2 and miR-217.

Having confirmed that miR-217 expression was negatively regulated by circ-TTBK2 expression, we further investigated whether miR-217 reversed circ-TTBK2-mediated promotion of glioma cells progression. The proliferation of glioma cells in the circ-TTBK2 (+)+miR-217 (−) group was significantly increased than that in the circ-TTBK2 (+)+miR-217 (+) group (Fig. 3i). Further, circ-TTBK2 introduction combined with miR-217 inhibition led to an increased cell number compared with circ-TTBK2 (+)+miR-217 (+) (Fig. 3j). Moreover, glioma cells treated with circ-TTBK2 (−)+miR-217 (+) exhibited higher ratio of apoptosis compared with cells treated with circ-TTBK2 (−)+miR-217 (−) (Fig. 3k). These results clarified that circ-TTBK2 promoted glioma cells malignant progression via reducing miR-217 expression.

### HNF1β was a target of miR-217, and was involved in circ-TTBK2- and miR-217-mediated modulation of glioma cells malignant progression

**Fig. 4:**
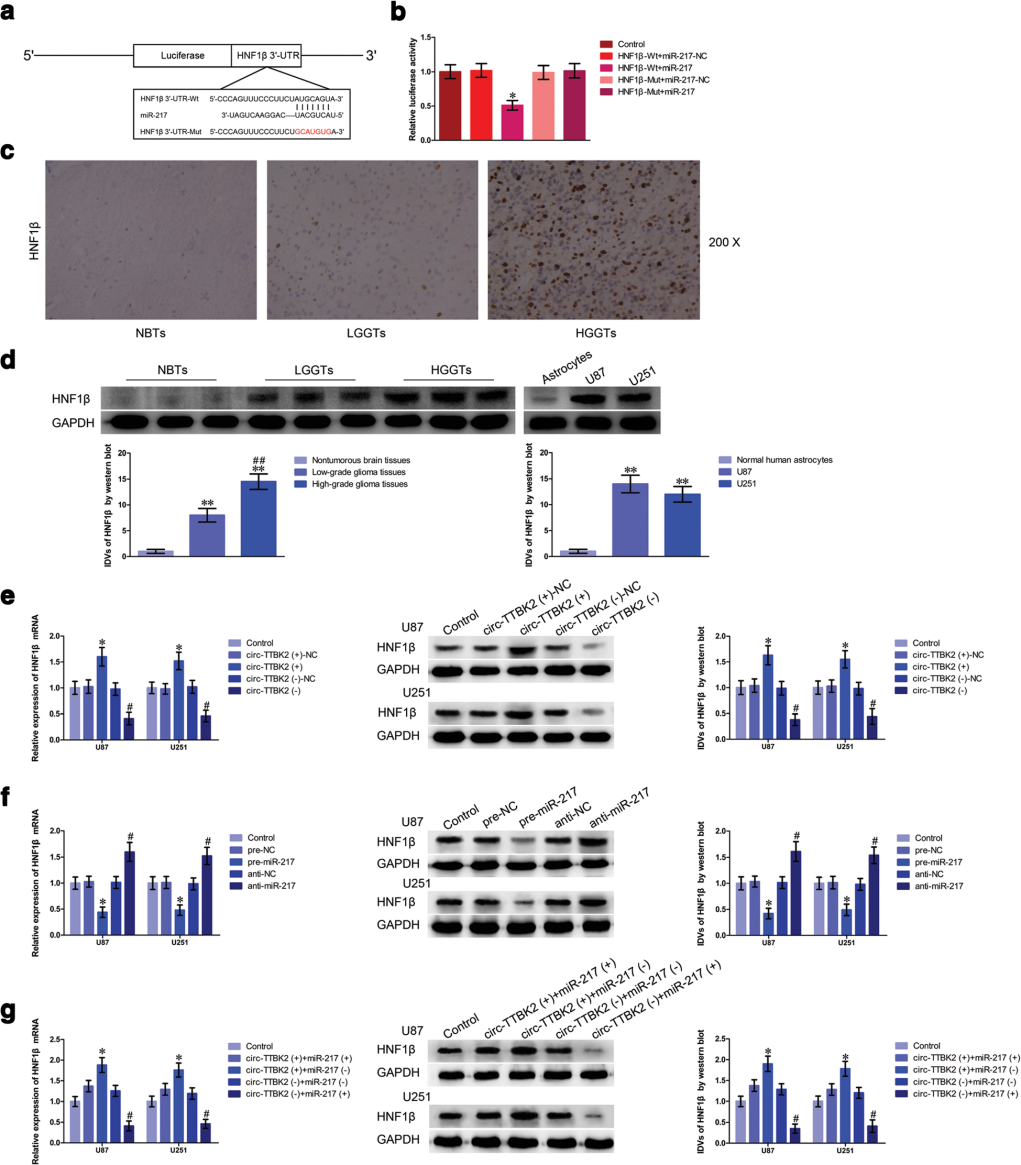
HNF1β was upregulated in glioma tissues and cell lines and was a target of miR-217; both circ-TTBK2 and miR-217 could modulate HNF1β expression. **a** The predicted miR-217 binding site in HNF1β (HNF1β-Wt) or and the designed mutant sequence (HNF1β-Mut) were indicated. **b** Luciferase reporter assay of HEK 293T cells co-transfected with HNF1β-Wt or HNF1β-Mut and miR-217 or the miR-217-NC (data are presented as the mean + SD (*n* = 5, each group). ^*^ *P* < 0.05 vs. HNF1β-Wt+miR-217-NC group). **c** Immunohistochemistry of HNF1β protein in nontumorous brain, low-grade glioma, and high-grade glioma tissues. Original magnification: 100×. Scale bar = 50 μm. **d** HNF1β protein expression levels in nontumorous brain tissues and glioma tissues using GAPDH as an endogenous control. Representative protein expression and their integrated density values (IDVs) of HNF1β in nontumorous brain tissues, low-grade glioma tissues (World Health Organization (WHO) I–II), and high-grade glioma tissues (WHO III-IV) are shown (data are presented as the mean + SD (*n* = 15, each group). ^**^ *P <* 0.01 vs. NBTs group; ^*##*^ *P <* 0.01 vs. low-grade glioma tissues group). HNF1β protein expression levels in astrocytes, U87, and U251 cells and using GAPDH as an endogenous control. Representative protein expression and their IDVs in human normal astrocytes, U87, and U251 are shown (data are presented as the mean + SD (*n* = 15, each group). ^**^ *P <* 0.01 vs. human normal astrocytes group). **e** qRT-PCR and western blot analysis for circ-TTBK2 regulated HNF1β expression in U87 and U251 cells. The relative expression of HNF1β mRNA was shown using GAPDH as an endogenous control. The IDVs of PIWIL4 was shown using GAPDH as an endogenous control (data are presented as the mean + SD (*n* = 5, each group). ^*^ *P <* 0.05 vs. circ-TTBK2 (+)-NC group; ^*#*^ *P <* 0.05 vs. circ-TTBK2 (−)-NC group). **f** qRT-PCR and western blot analysis for miR-217 regulated HNF1β expression in U87 and U251. The relative expression of HNF1β mRNA was shown using GAPDH as an endogenous control. The IDVs of HNF1β was shown using GAPDH as an endogenous control (data are presented as the mean + SD (*n* = 5, each group). ^*^ *P <* 0.05 vs. pre-NC group; ^*#*^ *P <* 0.05 vs. anti-NC group). **g** qRT-PCR and western blot analysis for circ-TTBK2 combined with miR-217 regulated HNF1β expression in U87 and U251. The relative expression of HNF1β mRNA was shown using GAPDH as an endogenous control. The IDVs of HNF1β was shown using GAPDH as an endogenous control (data are presented as the mean + SD (*n* = 5, each group). ^*^ *P <* 0.05 vs. circ-TTBK2 (+)+miR-217 (+) group; ^*#*^ *P <* 0.05 vs. circ-TTBK2 (−)+miR-217 (−) group)

Using bioinformatics databases (TargetScan, miRanda, and RNAhybrid), HNF1β was identified as a direct target of miR-217. The luciferase activity in the HNF1β-Wt+miR-217 group was significantly attenuated than that in the HNF1β-Wt+miR-217-NC group, while the luciferase activity in the HNF1β-Mut+miR-217 group was not affected (Fig. 4b). Accumulated evidence showed that HNF1β was upregulated in various tumors and exerted key roles in molecular events in cells. HNF1β was located to the nucleus and was positively correlated with the progression of glioma pathological grades (Fig. 4c). Furthermore, protein levels of HNF1β were significantly higher in low- and high-grade glioma tissues than those in normal brain tissues (Fig. 4d). In addition, HNF1β protein levels were upregulated in U87 and U251 cells compared with normal human astrocytes. Moreover, qRT-PCR and western blot were used to determine the expressions of HNF1β mRNA and protein in glioma cells treated with circ-TTBK2 inhibition and/or miR-217 overexpression. mRNA and protein expressions of HNF1β were robustly reduced than that in the circ-TTBK2 (−)-NC group (Fig. 4e). While mRNA and protein expressions of HNF1β were significantly decreased in the pre-miR-217 group compared with those in the pre-NC group (Fig. 4f). Moreover, mRNA and protein expressions of HNF1β were obviously upregulated in the circ-TTBK2 (+)+miR-217 (−) group than those in the circ-TTBK2 (+)+miR-217 (+) group (Fig. 4g).

### HNF1β promoted malignant progression of glioma cells and bound to the promoter of Derlin-1

**Fig. 5:**
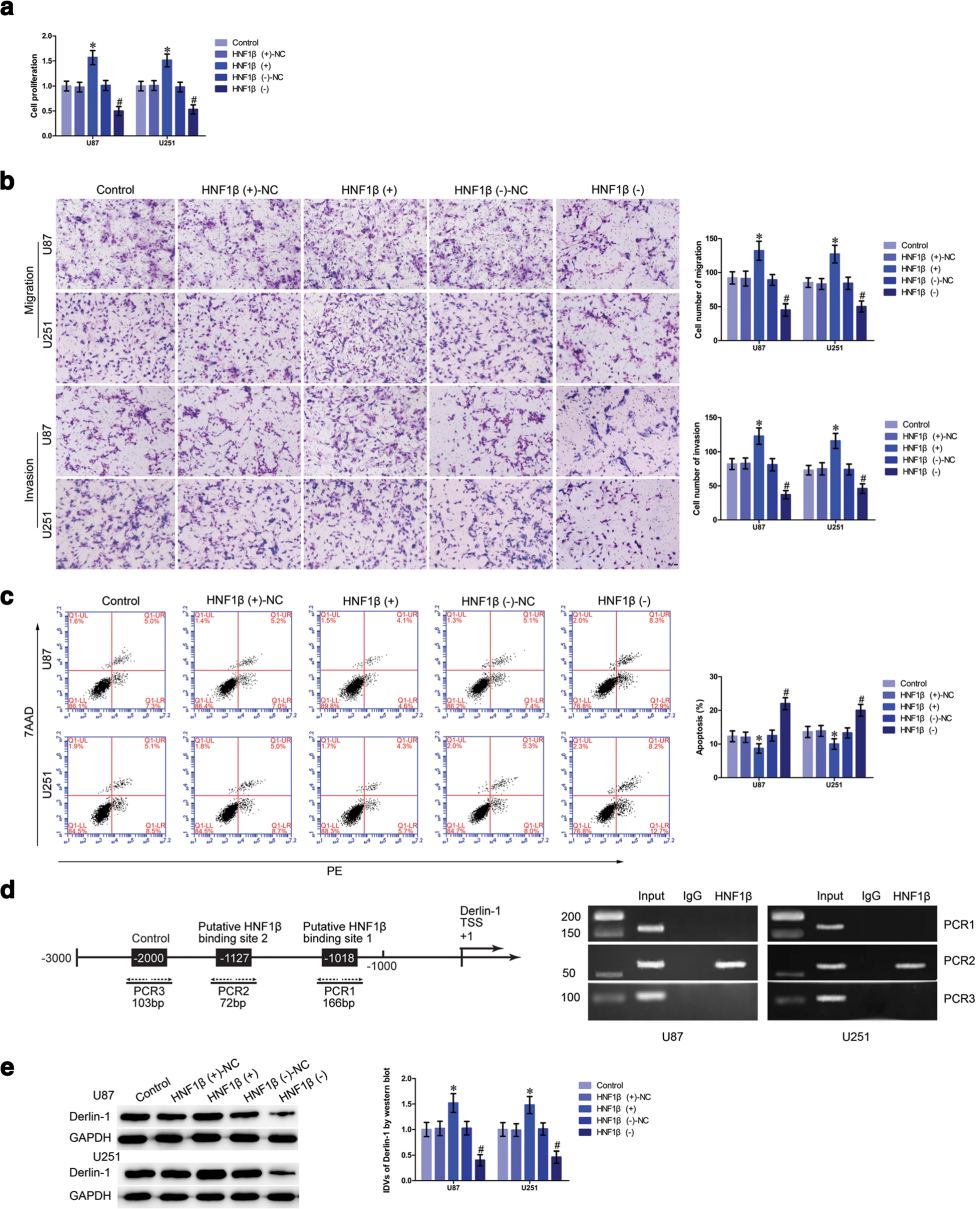
HNF1β exerted oncogenic role and could modulate Derlin-1 expression via binding to its promoter region in glioma cells. **a** CCK-8 assay was used to determine the proliferation effect of HNF1β on U87 and U251 cells. **b** Quantification number of migration and invasion cells with overexpression or inhibition of HNF1β. Representative images and accompanying statistical plots were presented. Scale bars represent 40 μm. **c** Flow cytometry analysis of U87 and U251 cells with the expression of HNF1β changed (data are presented as the mean + SD (*n* = 5, each group). ^*^ *P <* 0.05 vs. HNF1β (+)-NC group; ^*#*^ *P <* 0.05 vs. HNF1β (-)-NC group). **d** HNF1β bound to the promoters of Derlin-1 in U87 and U251 glioma cells. Transcription start site (TSS) was designated as +1. Putative HNF1β binding sites are indicated. Immunoprecipitated DNA was amplified by PCR. Normal rabbit IgG was used as a negative control. **e** Western blot analysis for HNF1β regulated IDVs of Derlin-1; they are shown using GAPDH as endogenous control. Data are presented as the mean + SD (*n* = 5, each group). ^*^ *P <* 0.05 vs. HNF1β (+)-NC group; ^*#*^ *P <* 0.05 vs. HNF1β (−)-NC group

Having confirmed that HNF1β was upregulated in glioma tissues and cells, effects of HNF1β overexpression or downregulation on glioma cells were investigated. The transfection efficiency of HNF1β was confirmed (Additional fie 3: Figure S1h). Cell proliferation in the HNF1β (+) group was significantly upregulated versus that in HNF1β (+)-NC group (Fig. 5a). Moreover, transwell assays showed that the migrating and invading cell numbers in the HNF1β (−) group were reduced compared with those in the HNF1β (-)-NC group (Fig. 5b). Flow cytometry analysis was used to measure whether HNF1β affected the apoptosis of glioma cells. Cells treated with HNF1β (+) exhibited lower apoptosis ratio than that in the HNF1β (+)-NC group (Fig. 5c).

HNF1β specifically binds to the “GTTAAT box” which is presented in promoters of target genes [28]. According to DBTSS HOME database, promoter sequence of Derlin-1 was set. By scanning the DNA sequence in the 2000 bp region upstream and 200 bp region downstream of the transcription start site (TSS), two putative HNF1β binding sites were identified. Chromatin immunoprecipitation (ChIP) assays were performed to determine whether HNF1β was directly associated with Derlin-1 promoters in U87 and U251 cells. As a negative control, 1000 bp upstream region of the putative HNF1β binding site was amplied using PCR, which was not expected to associate with HNF1β. There was a direct association of HNF1β with putative binding site 2 of Derlin-1 (Fig. 5d). And there were no interactions of HNF1β with the control region or putative binding site 1.

Further, Derlin-1 protein expression was detected in U87 and U251 cells treated with HNF1β (+) or HNF1β (-). Overexpressed HNF1β significantly increased Derlin-1 protein expression than that in the HNF1β (+)-NC group (Fig. 5e). These results demonstrated that HNF1β acted as an oncogene in glioma cells and could upregulate Derlin-1 expression by directly activating promoter of Derlin-1.

### Derlin-1 promoted cell proliferation, migration, and invasion, while inhibited apoptosis of glioma cells

**Fig. 6:**
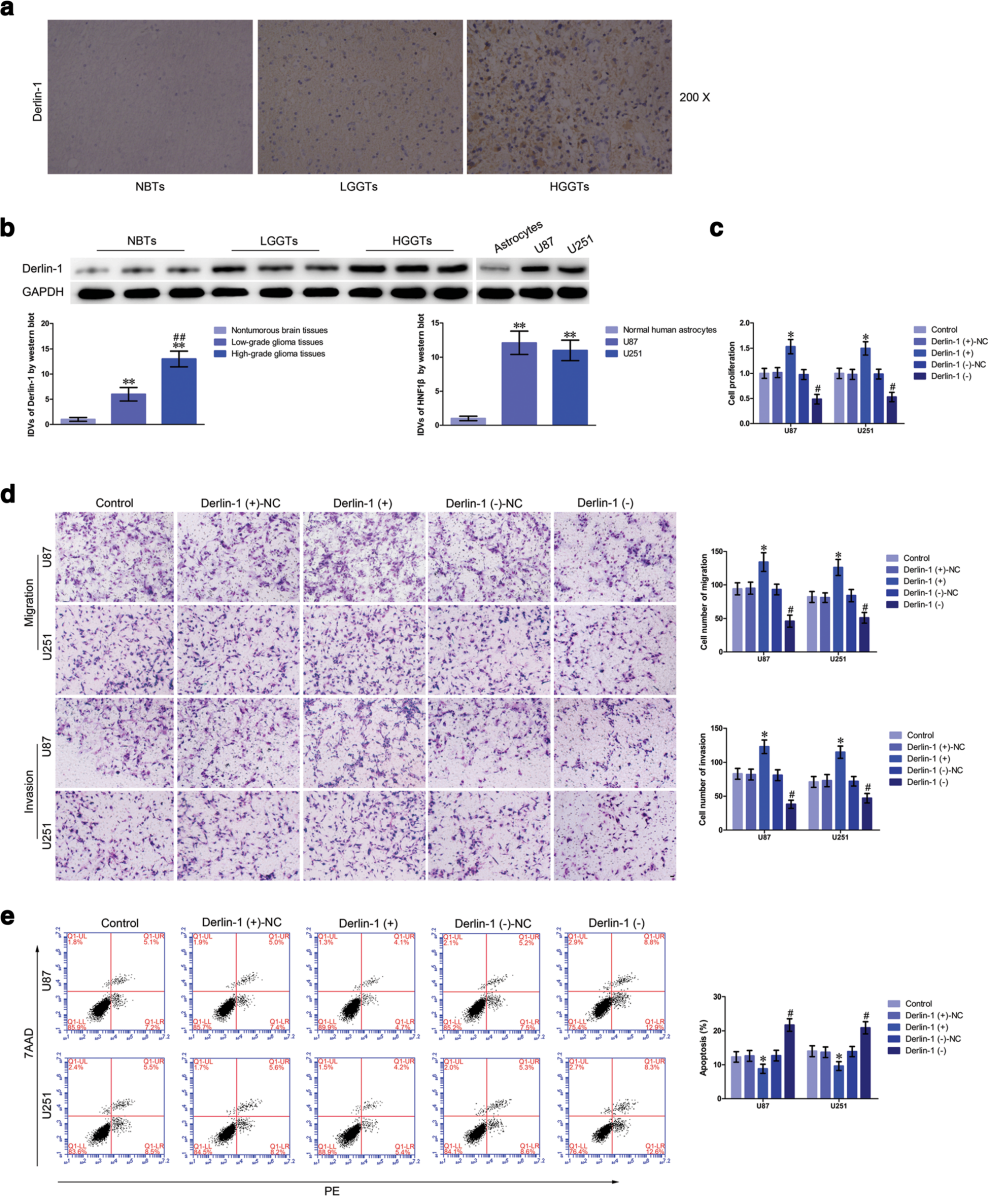
Derlin-1 was upregulated in glioma tissues and cell lines and played oncogenic role in glioma cells. **a** Immunohistochemistry of Derlin-1 protein in nontumorous brain, low-grade glioma, and high-grade glioma tissues. Original magnification: 100×. Scale bar = 50 μm. **b** Derlin-1 protein expression levels in nontumorous brain tissues and glioma tissues using GAPDH as an endogenous control. Representative protein expression and their integrated density values (IDVs) of Derlin-1 in nontumorous brain tissues, low-grade glioma tissues (World Health Organization (WHO) I–II), and high-grade glioma tissues (WHO III-IV) are shown (data are presented as the mean + SD (*n* = 15, each group). ^**^ *P <* 0.01 vs. NBTs group; ^*##*^ *P <* 0.01 vs. low-grade glioma tissues group). Derlin-1 protein expression levels in astrocytes, U87, and U251 cells and using GAPDH as an endogenous control. Representative protein expression and their IDVs in human normal astrocytes, U87, and U251 are shown (data are presented as the mean + SD (*n* = 15, each group). ^**^ *P <* 0.01 vs. human normal astrocytes group). **c** CCK-8 assay was used to measure the proliferation effect of Derlin-1 on U87 and U251 cells. **d** Quantification number of migration and invasion cells with reintroduction or knockdown of Derlin-1. Representative images and accompanying statistical plots were presented. Scale bars represent 40 μm. **e** Flow cytometry analysis of U87 and U251 cells with the expression of Derlin-1 changed. (Data are presented as the mean + SD (*n* = 5, each group). ^*^ *P <* 0.05 vs. Derlin-1 (+)-NC group; ^*#*^ *P <* 0.05 vs. Derlin-1 (−)-NC group)

Since Derlin-1 could be upregulated by HNF1β, we first examined the expression and function of Derlin-1 in glioma tissues and cells. Immunohistochemistry (IHC) results showed that Derlin-1 protein was located in cytoplasm and was positively correlated with the progression of glioma pathological grades (Fig. 6a). Moreover, Derlin-1 expression levels were obviously higher in low- and high-grade glioma tissues than in normal brain tissues. Similarly, Derlin-1 had a higher expression levels in U87 and U251 cells than those in human normal astrocytes (Fig. 6b). Further, effects of Derlin-1 on glioma cell biological behavior were determined. The transfection efficiency of Derlin-1 was determined by western blot (Additional file 1: Figure S1i). Overexpression of Derlin-1 led to an increased cell proliferation than that in the Derlin-1 (+)-NC group (Fig. 6c). Migrating and invading cells in the Derlin-1 (+) group were obviously increased compared with the Derlin-1 (+)-NC group (Fig. 6d). The apoptosis of cells treated with Derlin-1 (+) was impaired versus Derlin-1 (+)-NC group (Fig. 6e). These results showed that Derlin-1 functioned as an oncogene in glioma cells.

### Overexpressed miR-217 impaired HNF1β-induced promotion of glioma cell progression and regulated signaling pathways by targeting 3′-UTR of HNF1β

**Fig. 7:**
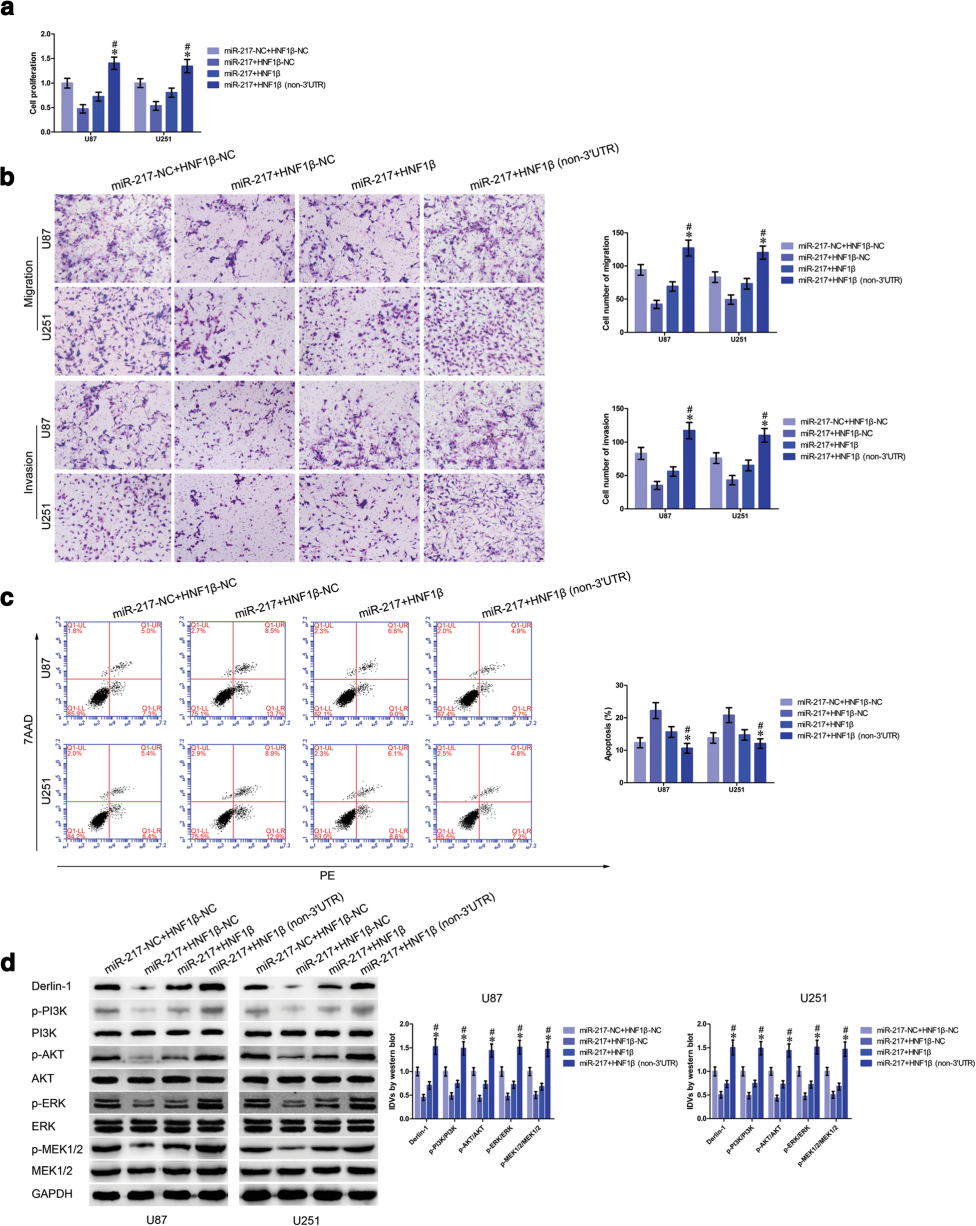
miR-217 inhibited glioma cell malignant progression by regulating PI3K/AKT and ERK pathways via targeting HNF1β 3′-UTR. **a** CCK-8 assay was applied to determine the proliferation effect of miR-217 and HNF1β on U87 and U251 cells (data are presented as the mean + SD (*n* = 5, each group). Data are presented as the mean + SD (*n* = 5, each group). ^*^ *P* < 0.05 vs. miR-217+HNF1β group; ^#^ *P* < 0.05 vs. miR-217+HNF1β-NC group. **b** Quantification of migration and invasion cells with the expression of miR-217 and HNF1β changed. Representative images and accompanying statistical plots were presented (data are presented as the mean + SD (*n* = 5, each group). ^*^ *P* < 0.05 vs. miR-217+HNF1β group; ^#^ *P* < 0.05 vs. miR-217+HNF1β-NC group. Scale bars represent 40 μm). **c** Flow cytometry analysis of U87 and U251 with the expression of miR-217 and HNF1β changed (data are presented as the mean + SD (*n* = 5, each group). ^*^ *P* < 0.05 vs. miR-217+HNF1β group; ^#^ *P* < 0.05 vs. miR-217+HNF1β-NC group). **d** Western blot analysis of p-PI3K, PI3K, p-AKT, AKT, p-ERK, ERK, p-MEK1/2, and MEK1/2 regulated by miR-217 and HNF1β in U87 and U251 cells, they are shown using GAPDH as endogenous control (data are presented as the mean + SD (*n* = 5, each group). ^*^ *P* < 0.05 vs. miR-217+HNF1β group; ^#^ *P* < 0.05 vs. miR-217+HNF1β-NC group)

The above results showed that circ-TTBK2 modulated HNF1β expression via regulating miR-217 expression, and HNF1β increased Derlin-1 expression by activating the promoter of Derlin-1. To clarify whether HNF1β reversed miR-217-induced impairment of malignant progression of glioma cells, we measured the biological behavior of glioma cells that stably expressed miR-217+HNF1β (non-3′UTR). Proliferation of glioma cells in the miR-217+HNF1β (non-3′UTR) group was significantly restored than that in the miR-217+HNF1β group (Fig. 7a). Furthermore, numbers of migrating and invading cells in the miR-217+HNF1β (non-3′UTR) group were increased versus those in the miR-217+HNF1β group (Fig. 7b). Moreover, glioma cells treated with miR-217+HNF1β (non-3′UTR) showed a lower apoptosis ratio than cells treated with miR-217+HNF1β (Fig. 7c).

As previously reported, Derlin-1 exerted oncogenic function by activating PI3K/AKT and ERK pathways; we next examined the expression of proteins involved in these signaling pathways. Derlin-1, p-PI3K, p-AKT, p-ERK, and p-MEK1/2 expression levels were significantly restored in the miR-217+HNF1β (non-3′UTR) group than those in the miR-217+HNF1β group (Fig. 7d).

### Knockdown of circ-TTBK2 combined with overexpression of miR-217 inhibited tumor growth and led to higher survival in nude mice

**Fig. 8:**
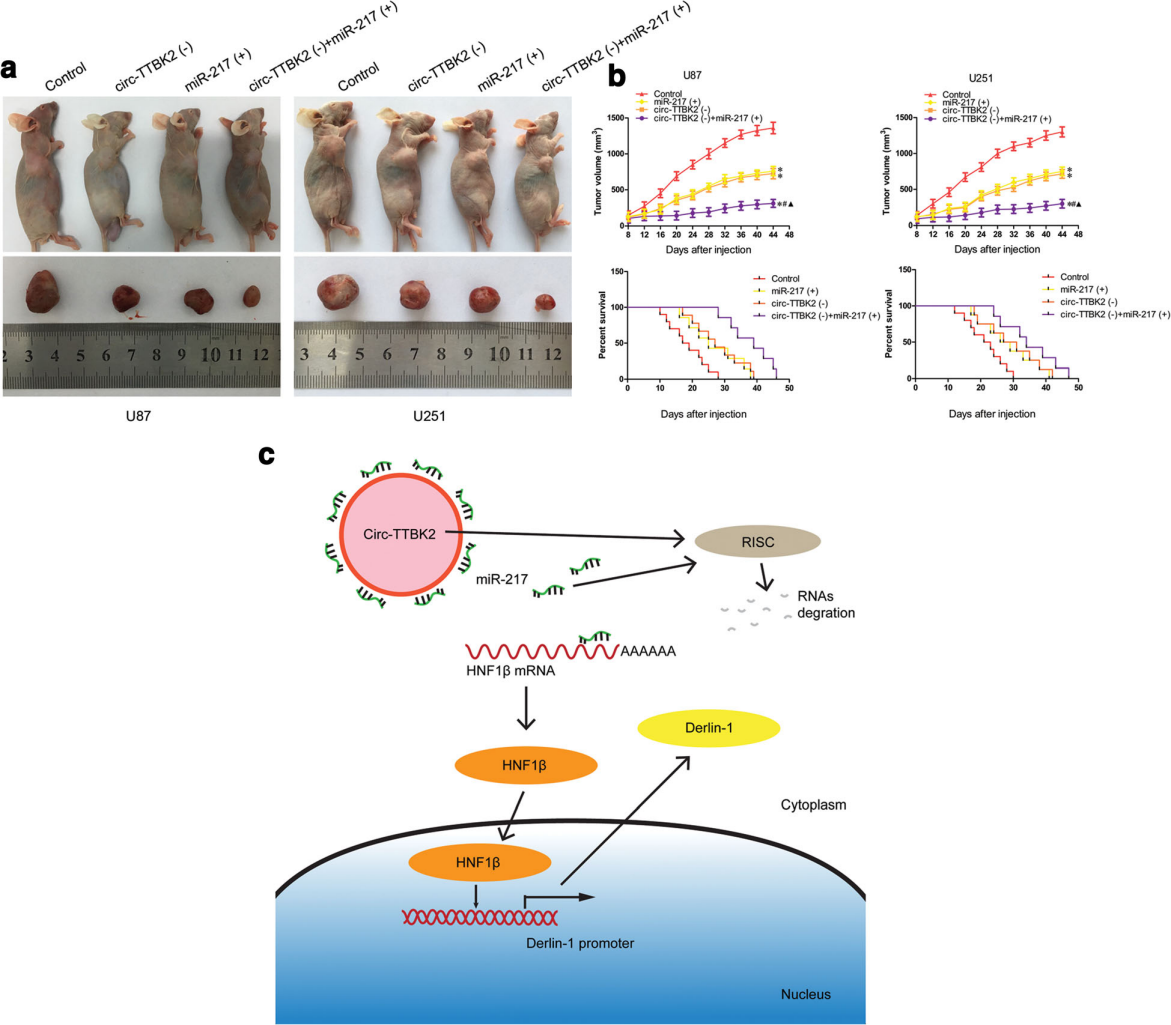
In vivo study and the schematic cartoon of the mechanism of circ-TTBK2. **a** The stable expressing cells were used for the in vivo study. The nude mice carrying tumors from respective groups were shown. The sample tumor from respective group was shown. **b** Tumor volume was calculated every 4 days after injection, and the tumor was excised after 44 days. ^*^ *P* < 0.05 vs. control group; ^#^ *P* < 0.05 vs. miR-217 (+) group; ^▲^ *P* < 0.05 vs. circ-TTBK2 (−) group. The survival curves of nude mice injected into the right striatum (*n* = 15). *P* < 0.05 (miR-217 (+) or circ-TTBK2 (−) vs. control group); *P* < 0.01 (circ-TTBK2 (−)+miR-217 (+) group vs. control group). **c** The schematic cartoon of the mechanism of circ-TTBK2 as an oncogene negative regulation of miR-217 in glioma cells

The in vivo experiment showed downregulated circ-TTBK2 or overexpressed miR-217 (Fig. 8b). Remarkably, knockdown of circ-TTBK2 combined with miR-217 overexpression resulted in the smallest tumor volume (Fig. 8b). The survival analysis demonstrated that in circ-TTBK2 inhibition, miR-217 reintroduction led to longer survival than the control group. And circ-TTBK2 inhibition combined with miR-217 overexpression produced the longest survival period (Fig. 8b).

## Discussion

In this study, we demonstrated that circ-TTBK2 was upregulated in glioma tissues and cell lines. Overexpression of circ-TTBK2 promoted glioma cells malignant progression. In contrary, miR-217 was downregulated in glioma tissues and cell lines. Restoration of miR-217 restrained glioma cells malignant progression. Moreover, miR-217 bound to circ-TTBK2 in a sequence-dependent manner and there was a reciprocal negative feedback between circ-TTBK2 and miR-217. Further, overexpression of circ-TTBK2 increased HNF1β expression via impairing miR-217 expression which negatively regulated HNF1β by targeting its 3′-UTR. HNF1β was upregulated in glioma tissues and cells and promoted cell proliferation, migration, and invasion, while inhibited apoptosis of glioma cells. Meanwhile, HNF1β enhanced the promoter activity and bound to the promoter of Derlin-1. In addition, Derlin-1, identified as an oncogene in glioma tissues and cells, was involved in the HNF1β-mediated promotion of glioma cells malignant progression. Overexpression of Derlin-1 facilitated malignancy of glioma cells. Mechanistically, PI3K/AKT and ERK pathways were involved in circ-TTBK2 regulated malignant progression of glioma cells. Remarkably, the in vivo study demonstrated that the inhibition of circ-TTBK2 and restoration of miR-217 exhibited the lowest tumor volume and the longest survival tumor-bearing nude mice.

Although circRNAs have been found decades ago, their novel functions have remained unclear until recently. Accumulated evidence indicated that dysregulated expression of circRNAs were ubiquitously in heterogeneous tumors and were involved in multiple cellular biological processes in tumor cells [29, 30]. Notwithstanding, mechanisms of circRNAs’ effect on tumor cells are anfractuous and unclear. As earlier reported, circ-Foxo3 expression was downregulated in several cancer cells than in non-cancer cells, and overexpressed circ-Foxo3 inhibited cell proliferation through binding to CDK2 and p21 [31]. Meanwhile, circ-Foxo3 expression level is upregulated in aged patient and mice tissues, overexpression of circ-Foxo3 contributes to the progression of senescence in mice Dox-induced cardiomyopathy cells by interacting with ID1, E2F1, FAK, and HIF1A [32]. Due to our preliminary result, circ-TTBK2 expression was upregulated in glioma tissues. Linear TTBK2 was first characterized as a kinase in brain cells that could phosphorylate Ser 208 and Ser 210 in tau protein [33]. Also, mutated TTBK2 contributed to spinocerebellar ataxia [34]. Remarkably, TTBK2 mRNA expression was not changed in glioma tissues and cells. Furthermore, neither circ-TTBK2 nor linear TTBK2 influenced respective expression level. Consistent with several previous findings, we concluded circ-TTBK2 was independent of linear TTBK2 [31, 35]. Moreover, enhanced circ-TTBK2 facilitated malignant progression of glioma cells. Therefore, circ-TTBK2 might be involved in the modulation of glioma cell biological behavior and exerted critical function in glioma progression.

Accumulated evidence confirmed that circRNAs may act as miRNAs sponges via binding to miRNAs and modulate their function [36]. For example, circ_005169 exerted oncogenic function via sponging miR-145 and increasing the expression of E2F5, BAG4, and FMNL2 in colorectal cancer cells [37]. Bioinformatics database (Starbase) showed that a putative binding site exists between circ-TTBK2 and miR-217. We further ascertained that miR-217 targeted circ-TTBK2-Wt. This indicated that circ-TTBK2 might sponge to miR-217 to modulate its function in glioma cell. Interestingly, two putative binding sites were identified in linear TTBK2. Our data showed that miR-217 targeted TTBK2-Wt1, this binding site was the same sequence as circ-TTBK2 harbored. However, the binding site falls within the CDS region of linear TTBK2 (not 3′ UTR). In addition, pre-miR-217 did not change the linear TTBK2 expression. This demonstrated that the binding site between TTBK2 and miR-217 was not functional. Further, we found that there was a reciprocal negative feedback between circ-TTBK2 and miR-217. The RIP assay results showed that circ-TTBK2 and miR-217 were presented in the RISC complex. This might partially explain why the expression of circ-TTBK2 and miR-217 were negatively correlated. We further investigated whether circ-TTBK2 exerted oncogenic function in glioma through regulating miR-217 and found that the restoration of miR-217 robustly reversed the circ-TTBK2-induced promotion of glioma cell malignant progression. These results demonstrated that miR-217 could target circ-TTBK2 in a sequence-specific manner, and there was a reciprocal repression process between circ-TTBK2 and miR-217.

Notoriously, miR-217 was downregulated in various tumors such as EOC and gastric cancer. Meanwhile, overexpressed miR-217 obviously inhibited cell proliferation, colony formation, and invasion, while promoted apoptosis of colorectal cancer cell via targeting AEG-1 3′-UTR [38]. Similarly, miR-217 was negatively correlated with malignant profiling and exerted tumor-suppressive function by restraining malignant biological behavior of human osteosarcoma cells [39]. Further, miR-217 expression was significantly lower in lung cancer tissues than in noncancerous tissues, and enhanced miR-217 inhibited cell proliferation, migration, and invasion, while induced apoptosis of SPC-A-1 and A549 cells via targeting KRAS [40]. Our data demonstrated that miR-217 expression was reduced in glioma tissues and cells. Also, overexpression of miR-217 impeded glioma cells malignancy in vitro and reduced tumor growth in vivo. These findings indicated that miR-217 exerted tumor-suppressive function in glioma cells.

HNF1β was first identified as a liver-specific transcription factor and contributed to the malignant progression of HCC. Recent studies showed that HNF1β functioned as an oncogene in various tumors. HNF1β is upregulated in human prostate cancer and favors cell proliferation and tumor progression [41, 42]. Also, HNF1β expression is increased in human pancreatic cancer and predicts poor survival [21]. Due to the oncogenetic role of HNF1β in various tumors, and the putative binding site between miR-217 and HNF1β predicted with bioinformatics databases, we hypothesized that HNF1β might be involved in circ-TTBK2/miR-217 regulation network. Luciferase assay result confirmed that HNF1β was a target of miR-217. Also, our data showed that HNF1β served as an oncogene in glioma cells. We next aimed to investigate whether HNF1β was involved in the circ-TTBK2-mediated regulation of glioma cell progression. As we expected, our results showed that circ-TTBK2 inhibition impaired HNF1β mRNA and protein expressions. Moreover, reintroduction of miR-217 decreased HNF1β mRNA and protein expressions by targeting its 3′-UTR. Additionally, overexpressed miR-217 reversed circ-TTBK2-induced promotion of HNF1β expression. These corroborated the hypothesis that HNF1β was involved in circ-TTBK2/miR-217 regulation network.

In most cases, HNF1β serves as an activator in the transcriptional regulation of targeted genes. Increasing reports showed that Derlin-1 was upregulated in various tumors. By analyzing the promoter sequence of Derlin-1, two putative HNF1β binding sites were identified. ChIP assays corroborate our hypothesis that HNF1β could directly bind to Derlin-1 promoter. Furthermore, we demonstrated that overexpressed HNF1β activated Derlin-1 expression. Similarly, HNF1β played key roles in the regulation of intracellular cholesterol storage and the level of free cholesterol via binding to and activating ACAT promoter [28]. Overexpressed HNF1β favors tumor progression and inhibits apoptosis of tumor cells via enforcing osteopontin expression, which harbors HNF1β binding sites in its promoter [43].

Notoriously, Derlin-1 acts as an oncogene in various tumors. Earlier study showed that Derlin-1 was involved in the TCL1-mediated contribution to progression of chronic lymphocytic leukemia in mice [44]. In addition, Derlin-1 is obviously upregulated in human lung cancer cells, and the inhibition of Derlin-1 attenuates p62 degradation, which leads to the blockage of tumor cell autophagy [45]. To describe the Derlin-1 profile in glioma tissues and cells, the expression and function of Derlin-1 were determined. Consistent with previously reported, our results demonstrated that Derlin-1 was located in the cytoplasm and had a high expression in glioma tissues and cells. Further, we found an enhanced expression of Derlin-1 that stimulated glioma cell malignant behaviors. More importantly, overexpression of Derlin-1 had been proved to have the ability to activate PI3K/AKT and ERK pathways in tumor cells, which are the key pathways directly related to proliferation, migration, invasion, and apoptosis [26, 27, 46, 47]. Given that Derlin-1 could be activated by HNF1β, and miR-217 modulated glioma cell progression via targeting 3′-UTR of HNF1β, we next sought to investigate whether Derlin-1 and the downstream pathways were involved in miR-217-induced blunting effect on glioma cells. First, we found that overexpression of HNF1β (without 3′-UTR) reversed inhibition on glioma cell malignant biological behavior induced by miR-217. Then, our results demonstrated that Derlin-1, p-PI3K, p-AKT, p-ERK, and p-MEK1/2 were distinctly restored when glioma cells were co-transfected with miR-217 and HNF1β (without 3′-UTR). These data provided a novel insight into the molecular mechanism of circ-TTBK2 and miR-217. The mechanism underlying tumorgenesis of human glioma cell lines by circ-TTBK2 is schematically presented in Fig. 8c.

## Conclusions

In summary, our study revealed that circ-TTBK2 inhibition restrained malignant progression of glioma cells by upregulating miR-217. MiR-217 exerted tumor-suppressive function through downregulating HNF1β. HNF1β activated Derlin-1 via binding to its promoter. More importantly, inhibition of circ-TTBK2/miR-217/HNF1β/Derlin-1 axis may be a potential therapeutic target for human gliomas.

## Methods

### Human tissues specimens

For determination of circ-TTBK2 and miR-217, clinical specimens were divided into five group: NBTs (normal brain tissues) (*n* = 11), grade I (*n* = 19), grade II (*n* = 19), grade III (*n* = 19), and grade IV (*n* = 19). For determination of HNF1β and Derlin-1, clinical specimens were divided into three groups: NBTs (*n* = 8), grade I–II glioma group (low-grade glioma tissues) (*n* = 16), and grade III–IV glioma group (high-grade glioma tissues) (*n* = 16) based on the WHO 2007 classification of tumors by two experienced neuropathologists. Normal brain tissues (NBTs) were collected from patients’ fresh autopsy material (donation from individuals who died in traffic accident and confirmed to be free of any prior pathologically detectable conditions) were used as negative control.

### Cell culture

Human U87 and U251 glioma cell lines and human embryonic kidney (HEK) 293T cells were purchased from the Shanghai Institutes for Biological Sciences Cell Resource Center. Primary normal human astrocytes (NHA) were purchased from the Sciencell Research Laboratories (Carlsbad, CA, USA). For details, see Additional file 3.

### Fluorescence in situ hybridization (FISH)

For identification of circ-TTBK2 and miR-217 rearrangement in glioma tissues, circ-TTBK2 probe (green-labeled, Biosense, Guangzhou, China) (5′ CAATCTTTCTCAATGGTCTGACGTCA 3′) and miR-217 probe (red-labeled, Exiqon, Copenhagen, Denmark) were used. In brief, slides were treated with PCR-grade proteinase-K (Roche Diagnostics, Mannheim, Germany) blocked after with prehybridization buffer (3% BSA in 4 × saline-sodium citrate, SSC). The hybridization mix was prepared with circ-TTBK2 probe or miR-217 probe in hybridization solution. Then the slides was washed with washing buffer; the sections were stained with anti-digoxin rhodamine conjugate (1:100, Exon Biotech Inc, Guangzhou, China) at 37 °C for 1 h away from light. The sections were stained with 4′,6-diamidino-2-phenylindole (DAPI, Beyotime, China) for nuclear staining subsequently. All fluorescence images (100×) were captured using a fluorescence microscope (Leica, Germany).

### Reverse transcription and quantitative real-time PCR

Trizol reagent (Life Technologies Corporation, Carlsbad, CA, USA) was used to extract total RNA from the clinical tissues and NHA, U87, and U251 cells. See also Additional file 3.

### Western blot

Western blot was performed as previously described [48]. See Additional file 3 for details and antibodies used.

### Cell transfections

Cell transfections were performed as previously described [48]. See also Additional file 3.

### Cell proliferation assay

Cell Counting Kit-8 assay (CCK-8, Dojin, Japan) was used to investigate glioma cell proliferation. Also, see Additional file 3.

### Migration and invasion assays

Twenty-four-well chambers with 8-μm pore size (Corning, USA) was used for migration and invasion determination of U87 and U251 cells in vitro. For details, see Additional file 3.

### Apoptosis analysis

Cell apoptosis was determined by Annexin V-PE/7AAD staining (Southern Biotech, Birmingham, AL, USA). See also Additional file 3.

### Reporter vectors construction and luciferase assays

Dual-luciferase assays were performed as previously described [48]. See Additional file 3.

### RNA immunoprecipitation

RNA immunoprecipitation was performed as previously described [48]. In brief, glioma cells were lysed by a complete RNA lysis buffer from an EZ-Magna RIP kit (Millipore, Billerica, MA) according to the manufacturer’s protocol. See also Additional file 3.

### Immunohistochemistry assays

The slides of human glioma tissue samples (4 μm thick) were dewaxed, rehydrated, and incubated in 0.3% H_2_O_2_ for 10 min to inhibit endogenous peroxidase activity before blocking with 10% normal goat serum (MXB, Fuzhou, China) for 30 min and incubating overnight at 4 °C with rabbit polyclonal antibody against HNF1β (1:150, SAB, Chicago, IL). Slides were washed with PBS three times and then incubated with biotinylated rabbit anti-rabbit IgG for 1 h at room temperature. After incubation with avidinbiotin-peroxidase complex for 10 min, samples were stained with 3, 3′-diaminobenzidine. Slides were imaged under a light microscope (Olympus, Japan) at 100× magnification.

### Chromatin immunoprecipitation assay

ChIP assay was conducted with Simple ChIP Enzymatic Chromatin IP Kit (Cell signaling Technology, Danvers, Massachusetts, USA) according to the manufacturer’s instruction as previously described [49]. In brief, glioma cells were fixed with 1% formaldehyde and collected in lysis buffer. Two percent aliquots of lysates were used as an input control and the remaining lysates were immunoprecipitated with normal rabbit IgG or HNF1β antibody. Immunoprecipitated DNA was amplified by PCR using their specific primers (as Additional file 4).

### Tumor xenografts in nude mice

The tumor xenograft experiment was performed as previously described [48]. Stable expression U87 and U251 cells were used for in vivo study. For details, see also Additional file 3.

### Statistical analysis

Data are presented as mean + standard deviation (SD). All statistical analyses were evaluated by SPSS 18.0 statistical software with the Student’s *t* test or one-way analysis of variance ANOVA. Differences were considered to be significant when *P* < 0.05. Corresponding significance levels were indicated in the figures.

## Additional files

Additional file 1: Figure S1. Circ-TTBK2 was resistant to RNase R treatment, and the transfection efficiency of each target. a and b Expression level of TTBK2 mRNA in glioma tissues and cells (data are presented as the mean + SD (*n* = 5, each group)). c Expression level of circ-TTBK2 in glioma cells with RNase R treatment (data are presented as the mean + SD (*n* = 5, each group), ^**^ *P* < 0.01 vs. control in normal human astrocytes group; ^##^ *P* < 0.01 vs. RNase R in Normal human astrocytes group). d Expression level of TTBK2 in glioma cells treated with RNase R (data are presented as the mean + SD (*n* = 5, each group), ^**^ *P* < 0.01 vs. control group respectively). e qRT-PCR was used to detect the transfection efficiency of circ-TTBK2 (data are presented as the mean + SD (*n* = 5, each group). ^**^ *P* < 0.01 vs. circ-TTBK2 (+)-NC group; ^##^ *P* < 0.01 vs. circ-TTBK2 (−)-NC group). f qRT-PCR was used to detect the transfection efficiency of sh-TTBK2 (data are presented as the mean + SD (*n* = 5, each group). ^**^ *P* < 0.01 vs. sh-NC group). g qRT-PCR was conducted to investigate the transfection efficiency of miR-217 (data are presented as the mean + SD (*n* = 5, each group). ^**^ *P* < 0.01 vs. pre-NC group; ^##^ *P* < 0.01 vs. anti-NC group). h Western blot was used to investigate the transfection efficiency of HNF1β (data are presented as the mean + SD (*n* = 5, each group). ^**^ *P* < 0.01 vs. HNF1β (+)-NC group; ^##^ *P* < 0.01 vs. HNF1β (−)-NC group). i Western blot was used to investigate the transfection efficiency of Derlin-1 (data are presented as the mean + SD (*n* = 5, each group). ^**^ *P* < 0.01 vs. Derlin-1 (+)-NC group; ^##^ *P* < 0.01 vs. Derlin-1 (−)-NC group). (TIF 781 kb)
Additional file 2: Figure S2. Correlation between miR-217 and TTBK2. a qRT-PCR was conducted to detect expression levels of TTBK2 in glioma cell treated with circ-TTBK2 (+) and circ-TTBK2 (−) (*n* = 5, each group). b Expression level of circ-TTBK2 in cells treated with sh-TTBK2 (*n* = 5, each group). c Expression level of TTBK2 in cells treated with pre-miR-217 and anti-miR-217 (*n* = 5, each group). d The predicted two miR-217 binding sites in TTBK2 (TTBK2-Wt and TTBK2-Wt1) and the designed mutant sequences (TTBK2-Mut and TTBK2-Mut1) were indicated. e and f Luciferase reporter assays of HEK 293T cells co-transfected with TTBK2-Wt (or TTBK2-Wt1) or TTBK2-Mut (or TTBK2-Mut1), and miR-217 or the miR-217-NC (*n* = 5, each group). (TIF 408 kb)
Additional file 3: Additional materials and methods. (DOCX 32 kb)
Additional file 4: Primers used for chromatin immunoprecipitation assay. (XLSX 9 kb)

## Abbreviations

circRNAs: Circular RNAs
miRNA: MicroRNA
ncRNAs: Non-coding RNAs
TTBK2: Tau tubulin kinase 2
EOC: Epithelial ovarian cancer
HNF1β: Hepatocyte nuclear factor-1beta
HCC: Hepatocellular carcinoma
ER: Endoplasmic reticulum
RISC: RNA-induced silencing complex
FISH: Fluorescence in situ hybridization
qRT-PCR: Quantative Real-time PCR
RIP: RNA-binding protein immunoprecipitation
ChIP: Chromatin immunoprecipitation
