# Supplementary figures and images for: TTBK2 circular RNA promotes glioma malignancy by regulating miR-217/HNF1β/Derlin-1 pathway
Source: J Hematol Oncol. 2017 Feb 20;10:52. doi: 10.1186/s13045-017-0422-2 (PMC5319142; doi:10.1186/s13045-017-0422-2)

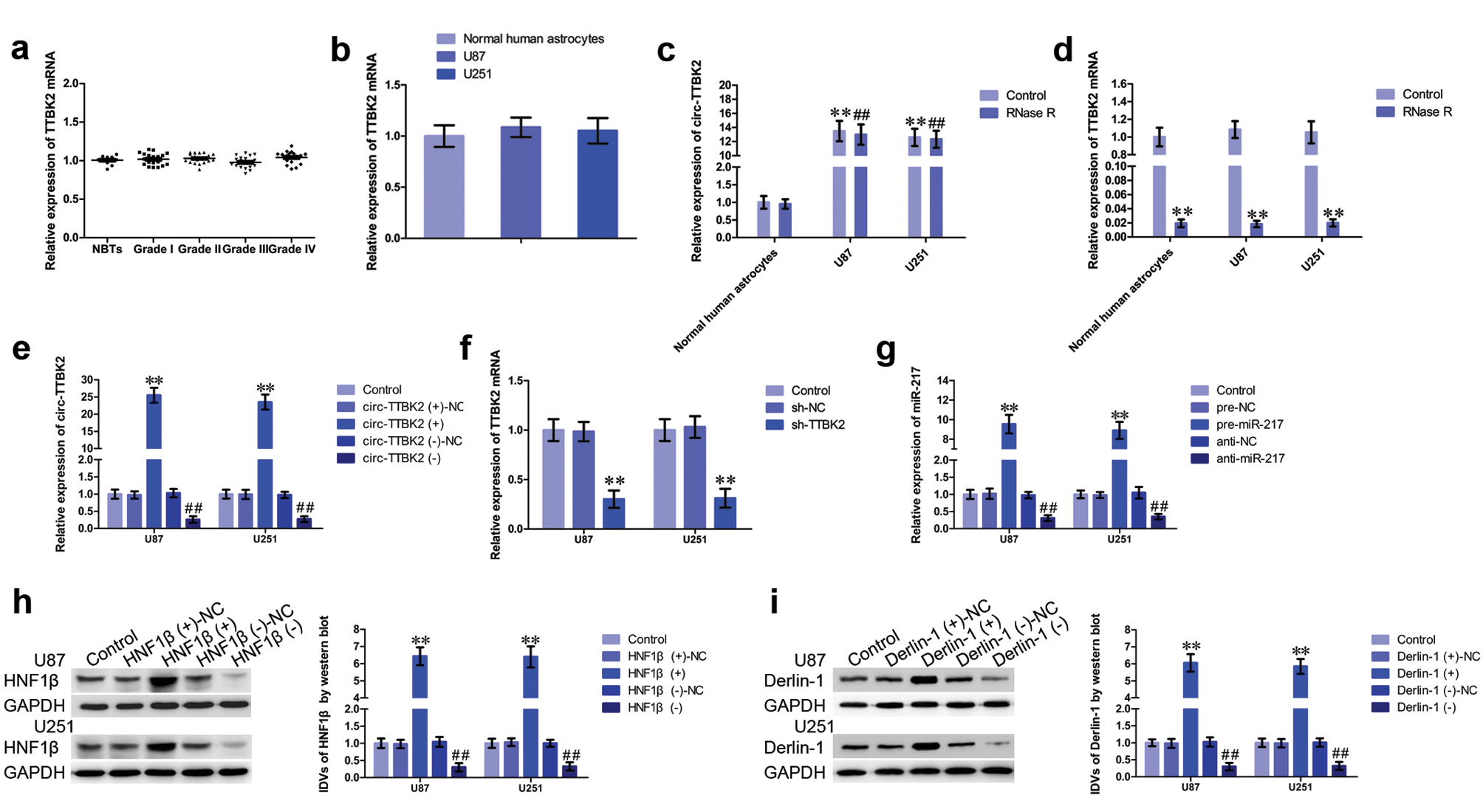

Supplement: Additional file 1: Figure S1. — Circ-TTBK2 was resistant to RNase R treatment, and the transfection efficiency of each target. a and b Expression level of TTBK2 mRNA in glioma tissues and cells (data are presented as the mean + SD (n = 5, each group)). c Expression level of circ-TTBK2 in glioma cells with RNase R treatment (data are presented as the mean + SD (n = 5, each group), ** P < 0.01 vs. control in normal human astrocytes group; ## P < 0.01 vs. RNase R in Normal human astrocytes group). d Expression level of TTBK2 in glioma cells treated with RNase R (data are presented as the mean + SD (n = 5, each group), ** P < 0.01 vs. control group respectively). e qRT-PCR was used to detect the transfection efficiency of circ-TTBK2 (data are presented as the mean + SD (n = 5, each group). ** P < 0.01 vs. circ-TTBK2 (+)-NC group; ## P < 0.01 vs. circ-TTBK2 (−)-NC group). f qRT-PCR was used to detect the transfection efficiency of sh-TTBK2 (data are presented as the mean + SD (n = 5, each group). ** P < 0.01 vs. sh-NC group). g qRT-PCR was conducted to investigate the transfection efficiency of miR-217 (data are presented as the mean + SD (n = 5, each group). ** P < 0.01 vs. pre-NC group; ## P < 0.01 vs. anti-NC group). h Western blot was used to investigate the transfection efficiency of HNF1β (data are presented as the mean + SD (n = 5, each group). ** P < 0.01 vs. HNF1β (+)-NC group; ## P < 0.01 vs. HNF1β (−)-NC group). i Western blot was used to investigate the transfection efficiency of Derlin-1 (data are presented as the mean + SD (n = 5, each group). ** P < 0.01 vs. Derlin-1 (+)-NC group; ## P < 0.01 vs. Derlin-1 (−)-NC group). (TIF 781 kb) [file 13045_2017_422_MOESM1_ESM.tif]

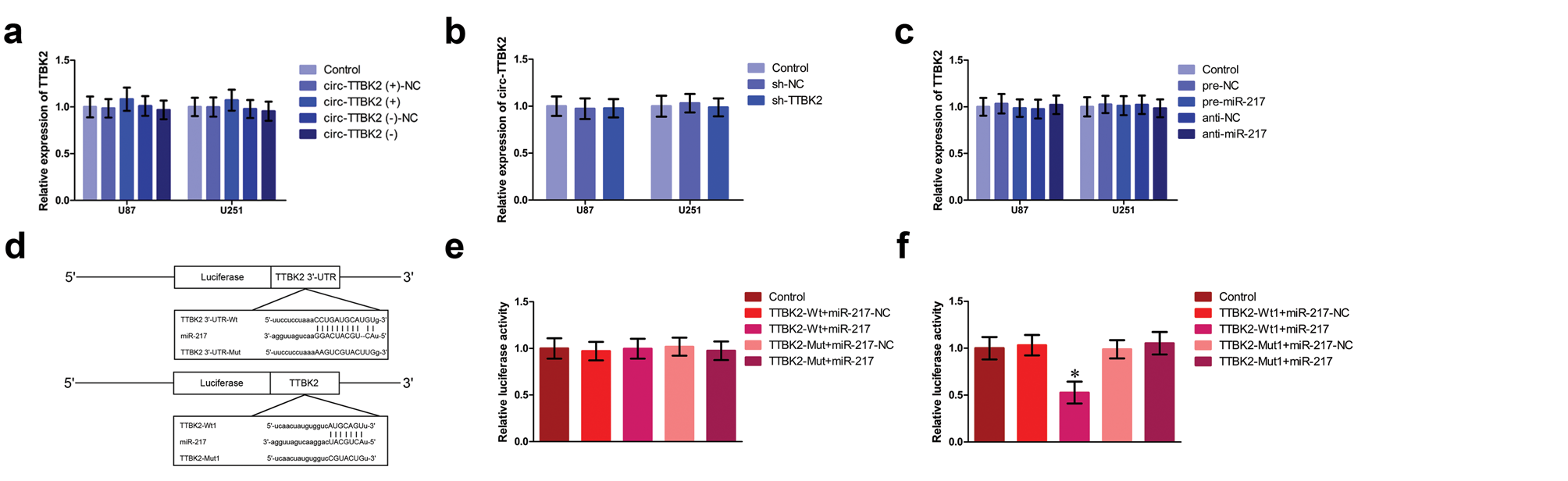

Supplement: Additional file 2: Figure S2. — Correlation between miR-217 and TTBK2. a qRT-PCR was conducted to detect expression levels of TTBK2 in glioma cell treated with circ-TTBK2 (+) and circ-TTBK2 (−) (n = 5, each group). b Expression level of circ-TTBK2 in cells treated with sh-TTBK2 (n = 5, each group). c Expression level of TTBK2 in cells treated with pre-miR-217 and anti-miR-217 (n = 5, each group). d The predicted two miR-217 binding sites in TTBK2 (TTBK2-Wt and TTBK2-Wt1) and the designed mutant sequences (TTBK2-Mut and TTBK2-Mut1) were indicated. e and f Luciferase reporter assays of HEK 293T cells co-transfected with TTBK2-Wt (or TTBK2-Wt1) or TTBK2-Mut (or TTBK2-Mut1), and miR-217 or the miR-217-NC (n = 5, each group). (TIF 408 kb) [file 13045_2017_422_MOESM2_ESM.tif]
