# Supplementary material for: TTBK2 circular RNA promotes glioma malignancy by regulating miR-217/HNF1β/Derlin-1 pathway
Source: J Hematol Oncol. 2017 Feb 20;10:52. doi: 10.1186/s13045-017-0422-2 (PMC5319142; doi:10.1186/s13045-017-0422-2)
Supplement: Additional file 3: — Additional materials and methods. (DOCX 32 kb) [file 13045_2017_422_MOESM3_ESM.docx]

**Supplementary Methods**

**Cell culture**

U87 glioma cells and HEK-293T cells were grown in Dulbecco's modified Eagle medium (DMEM)/high glucose supplemented with 10% fetal bovine serum (FBS, Gibco, Carlsbad, CA, USA). U251 cells were grown in DMEM/F12 medium supplemented with 10% FBS. NHA was cultured under the instructed condition by the manufacturer. All cells were maintained in standard conditions (37 °C, 5% CO_2_).

**Microarray analysis**

Microarray analysis was performed as previously reported [[1](#_ENREF_1)]. The microarray hybridization and the data collection were performed with the help of KangChen Bio-tech, Shanghai, China. CircRNAs (fold changes ≥ 2.0 and P-values < 0.05) were differentially expressed with statistical significance.

**Reverse transcription and quantitative real-time PCR (qRT-PCR)**

One Step PrimeScript™ RT-PCR Kits (Perfect Real Time) (Takara, RR064A, Japan) were used for measurement of circ-TTBK2. In addition, RNase-R was used to confirm the existent of circ-TTBK2, and eliminated the influence of liner RNAs. The primers of circ-TTBK2 and GAPDH synthesized from Takara (Japan), circ-TTBK2: forward 5′-AGTGCAACATTTTCCCTGGTG-3′, reverse 5′- GCTTGATTTTGGCTTGGCTC-3′, probe FAM+CCCCAATCTTTCTCAATGGTCTGACG+BHQ1, GAPDH: forward 5′-GGACCTGACCTGCCGTCTAG-3′, reverse 5′-TAGCCCAGGATGCCCTTGAG-3′, probe FAM+CCTCCGACGCCTGCTTCACCACCT+Eclipse. TaqMan MicroRNA Reverse Transcription kit and High Capacity cDNA Reverse Transcription Kit were used for miRNA and mRNA reverse transcription, respectively (Applied Biosystems, Foster City, CA, USA). The primers of TTBK2, HNF1β, Derlin-1, GAPDH, hsa-miR-217 and U6 were synthesized from the Applied Biosystems, TTBK2: forward 5′-GGATGTTTGGGAAGAGATGG-3′, reverse 5′-AGGCTTGGAGCATTGAGAAG-3′, HNF1β: forward 5′-ACACACCTCCCATCCTCAAG-3′, reverse 5′-CATTTTAGCAGCCCTCCAAG-3′, Derlin-1: forward 5′-CTCTGCCCTTGACAGGTTCT-3′, reverse 5′-TCTTCTCCCCCTATTGCTACA-3′, GAPDH: forward 5′-AAATCCCATCACCATCTTCCAG-3′, reverse 5′-TGATGACCCTTTTGGCTCCC-3′. Quantitative real-time PCR (qRT-PCR) was conducted using TaqMan Universal Master Mix II with TaqMan microRNA assays of miR-217 and U6 or TaqMan gene expression assays of HNF1β, Derlin-1 and GAPDH (Applied Biosystems, Foster City, CA, USA) using the ABI 7500 Fast Real-Time PCR System (Applied Biosystems). Expressions were normalized to endogenous controls and fold change was determined as 2^−ΔΔCt^ in gene expression.

**Western blot**

RIPA buffer with protease inhibitors (Beyotime Institute of Biotechnology) was used to extract total proteins on ice form the cells. Then the proteins were electrophoretically transferred to PVDF membranes. Membranes were incubated in TBS Blotto A Blocking Reagent (Santa Cruz Biotechnology) for 2 hours at room temperature and then incubated with primary antibodies as follows: HNF1β (1:500, Santa Cruz Biotechnology), Derlin-1 (1:5,000, Abcam, UK), p-PI3K (1:500, Bioworld, MN), PI3K (1:1,000, CST, EUGENE), p-AKT (1:2,000, CST, EUGENE), AKT (1:2,000, CST, EUGENE), p-ERK (1:1,000, CST, EUGENE), ERK (1:1,000, CST, EUGENE), p-MEK1/2 (1:1,000, CST, EUGENE), MEK1/2 (1:1,000, CST, EUGENE), and GAPDH (1:1,000, Santa Cruz Biotechnology), followed by incubation with appropriate correlated HRP-conjugated secondary antibody. Then the membranes were incubated with secondary antibodies (Santa Cruz Biotechnology) at room temperature for 2 hours. Immunoblots were visualized by enhanced chemiluminescence (ECL kit, Santa Cruz Biotechnology) and scanned using ChemImager 5,500 V2.03 software. The relative integrated density values (IDVs) were calculated based on GAPDH as an internal control.

**Cell transfections**

Circ-TTBK2 full length (circ-TTBK2 (+)) plasmid, short-hairpin circ-TTBK2 (circ-TTBK2 (-)) plasmid and their respective non-targeting sequence (negative control, NC) (circ-TTBK2 (+)-NC or circ-TTBK2 (-)-NC); short-hairpin TTBK2 (sh-TTBK2) plasmids and the respective non-targeting sequence (sh-NC) were constructed (Geenseed Biotech Co, Guangzhou, China). Consistent with previously reported, circ-TTBK2 plasmid contained a front circular frame and a back circular frame, the front circular frame contains the endogenous flanking genomic sequence referring to 226 bp upstream, and the back circular frame contains part of the inverted upstream sequence [[1](#_ENREF_1), [2](#_ENREF_2)]. The sequence for shRNA targeting circ-TTBK2: 5′ GGTGACGTCAGACCATTGAGA 3′. The sequence for shRNA targeting TTBK2: 5′ GCATCTTTCTAGACCATATCT 3′. miR-217 agomir (pre-miR-217), miR-217 antagomir (anti-miR-217) and their respective non-targeting sequence (negative control, NC) (pre-NC or anti-NC) were synthesized (GenePharma, Shanghai, China). HNF1β full length (with 3′-UTR) (HNF1β (+) or HNF1β) plasmid, short-hairpin HNF1β (HNF1β (-)) plasmid, HNF1β (without 3′-UTR) (HNF1β (non-3′UTR)) plasmid and their respective non-targeting sequence (negative control, NC) (HNF1β (+)-NC or HNF1β (-)-NC), Derlin-1 full length (Derlin-1 (+)) plasmid, short-hairpin Derlin-1 (Derlin-1 (-)) plasmid, and their respective non-targeting sequence (negative control, NC) (Derlin-1 (+)-NC or Derlin-1 (-)-NC) were synthesized (Life technology, MA, USA). The sequence for shRNA targeting HNF1β: 5′ GGAATGCAACAGGGCAGAATG 3′. The sequence for shRNA targeting Derlin-1: 5′ GCTCCTCTTTAACTGGATTTG 3′. Cells were seeded into 24-well plates (Corning) when they were at 50-70% confluence and then transfected using Lipofectamine 3000 reagent (Life Technologies Corporation, Carlsbad, CA, USA). G418 was used to select the applicable stably transfected cells. The over-expression and the silence efficiency were determined by qRT-PCR. To explore the effect of circ-TTBK2 on glioma, cells were divided into five groups: Control group, circ-TTBK2 (+)-NC group (tansfected with circ-TTBK2 (+)-NC plasmid), circ-TTBK2 (+) group (tansfected with circ-TTBK2 full length plasmid), circ-TTBK2 (-)-NC group (tansfected with circ-TTBK2 (-)-NC plasmid) and circ-TTBK2 (-) (transfected with short-hairpin circ-TTBK2 (-) plasmid) group. Similarly, to investigate the effect of miR-217 on glioma, cells were divided into five groups: Control group, pre-NC group (transfected with negative control), pre-miR-217 group (transfected with miR-217 agomir), anti-NC group (transfected with negative control) and anti-miR-217 (transfected with miR-217 antagomir). To determine the effect of HNF1β on glioma, cells were divide into five groups: Control group, HNF1β (+)-NC group (transfected with empty plasmid), HNF1β (+) group (transfected with HNF1β full length plasmid), HNF1β (-)-NC group (transfected with empty plasmid) and HNF1β (-) group (transfected with short-hairpin HNF1β plasmid). In addition, to study the effect of Derlin-1 on glioma, cells were divided into five groups: Control group, Derlin-1 (+)-NC group (transfected with empty plasmid), Derlin-1 (+) group (transfected with Derlin-1 full length plasmid), Derlin-1 (-)-NC group (transfected with empty plasmid) and Derlin-1 (-) group (transfected with short-hairpin Derlin-1 plasmid). Further, to explore the underlying mechanism of circ-TTBK2 regulated the malignant progression of glioma cells via impairing miR-217, cells were divided into five groups: Control group, circ-TTBK2 (+)+miR-217 (+) group (circ-TTBK2 (+) stable expressing cells co-transfected with pre-miR-217), circ-TTBK2 (+)+miR-217 (-) group (circ-TTBK2 (+) stable expressing cells co-transfected with anti-miR-217), circ-TTBK2 (-)+miR-217 (-) group (circ-TTBK2 (-) stable expressing cells co-transfected with anti-miR-217) and circ-TTBK2 (-)+miR-217 (+) group (circ-TTBK2 (-) stable expressing cells co-transfected with pre-miR-217). Furthermore, to determine miR-217 restrained the malignant progression of glioma cells via targeting HNF1β 3′-UTR, cells were dived into four groups: miR-217-NC+HNF1β-NC group (pre-NC stable expressing cells co-transfected with HNF1β-NC plasmid), miR-217+HNF1β-NC group (pre-miR-384 stable expressing co-transfected with HNF1β-NC), miR-217+HNF1β group (pre-miR-384 stable expressing co-transfected with HNF1β (+)) and miR-217+ HNF1β (non-3′UTR) group (pre-miR-217 stable expression transfected with HNF1β (without 3′-UTR) plasmid).

**Cell proliferation assay**

U87 and U251 glioma cells (2000 cells per well) were plated in 96-well plates. After cells were transfected 72h, 10 μL of CCK-8 solution was added into each well and cells were incubated for 2 h at 37 °C. The absorbance was measured at 450 nm using the SpectraMax M5 microplate reader (Molecular Devices, USA).

**Migration and invasion assays**

Cells were resuspended in 100 μL serum-free medium at a density of 1×10^5^/mL and seeded in the upper chamber for cell migration assay (or pre-coated with 500 ng/ml Matrigel solution (BD, Franklin Lakes, NJ, USA) for cell invasion assay). After incubation for 48 h, the cells on the upper membrane surface were physically removed. Cells that had migrated or invaded to the lower side of the membrane were fixed with methanol and stained with 10% Giemsa (Dinguo, China). Five randomly fields were selected to count cells for statistics under a microscope and photographs were taken.

**Apoptosis analysis**

After washing with 4 °C PBS twice, cells were collected and stained with Annexin V-PE/7AAD according to the manufacturer’s instruction. Then the cells were analyzed by flow cytometry (FACScan, BD Biosciences) and apoptotic fractions were investigated by CELL Quest 3.0 software.

**Reporter vectors construction and luciferase assays**

The potential miR-217 binding sites of circ-TTBK2, TTBK2 and HNF1β 3′-UTR sequences were amplified by PCR and cloned into a pmirGlo Dual-luciferase miRNA Target Expression Vector (Promega, Madison, WI, USA) to construct luciferase reporter vector (circ-TTBK2-Wt, TTBK2-Wt, TTBK2-Wt1 and HNF1β-Wt) (Geenseed Biotech Co, Guangzhou, China). The sequence of putative binding site was replaced as indicated (circ-TTBK2-Mut, TTBK2-Mut, TTBK2-Mut1 and HNF1β-Mut) to mutate the putative binding site of circ-TTBK2, TTBK2 or HNF1β. HEK-293T cells were seeded in 96-well plates and the cells were co-transfected with circ-TTBK2-Wt (or circ-TTBK2-Mut), TTBK2-Wt (or TTBK2-Mut), TTBK2-Wt1 (or TTBK2-Mut1) or HNF1β-Wt (or HNF1β-Mut) and miR-217 or miR-217-NC plasmids when they reached 50-70% confluence. The luciferase activities were detected at 48h after transfection by Dual-Luciferase reporter assay kit (Promega). The cells were divided into five groups respectively: Control group, circ-TTBK2-Wt+miR-217-NC (transfected with circ-TTBK2-Wt and pre-miR-217-NC), circ-TTBK2-Wt+miR-217 group (transfected with circ-TTBK2-Wt and pre-miR-217), circ-TTBK2-Mut+miR-217-NC group (transfected with circ-TTBK2-Mut and pre-miR-217-NC), circ-TTBK2-Mut+miR-217 group (transfected with circ-TTBK2-Mut and pre-miR-217); Control group, TTBK2-Wt+miR-217-NC (transfected with TTBK2-Wt and pre-miR-217-NC), TTBK2-Wt+miR-217 group (transfected with TTBK2-Wt and pre-miR-217), TTBK2-Mut+miR-217-NC group (transfected with TTBK2-Mut and pre-miR-217-NC), TTBK2-Mut+miR-217 group (transfected with TTBK2-Mut and pre-miR-217); Control group, TTBK2-Wt1+miR-217-NC (transfected with TTBK2-Wt1 and pre-miR-217-NC), TTBK2-Wt1+miR-217 group (transfected with TTBK2-Wt1 and pre-miR-217), TTBK2-Mut1+miR-217-NC group (transfected with TTBK2-Mut1 and pre-miR-217-NC), TTBK2-Mut1+miR-217 group (transfected with TTBK2-Mut1 and pre-miR-217); Control group, HNF1β-Wt+miR-217-NC (transfected with HNF1β-Wt and pre-miR-217-NC), HNF1β-Wt+miR-217 group (transfected with HNF1β-Wt and pre-miR-217), HNF1β-Mut+miR-217-NC group (transfected with HNF1β-Mut and pre-miR-217-NC), HNF1β-Mut+miR-217 group (transfected with HNF1β-Mut and pre-miR-217).

**RNA immunoprecipitation**

Whole cell lysate of the control groups and anti-miR-217 groups were incubated with RIP immunoprecipitation buffer containing magnetic beads conjugated with human anti-Argonaute2 (Ago2) antibody (Millipore), and NC normal mouse IgG (Millipore). Samples were incubated with Proteinase K buffer and then immunoprecipitated RNA was isolated. Further, purified RNA was collected and analyzed by qRT-PCR to validate the presence of the binding targets using respective primers mentioned earlier.

**Tumor xenografts in nude mice**

Lentivirus encoding miR-217 was generated using pLenti6.3/V5eDEST Gateway Vector Kit (Life Technologies). The miR-217 and short-hairpin RNA targeting human circ-TTBK2 were ligated into the pLenti6.3/V5eDEST vector and LV3-CMV-GFP-Puro vector (GenePharma), respectively. Further, pLenti6.3/V5eDEST-miR-217 and LV3-CMV-GFPPuro-sh-circ-TTBK2 vectors were generated. The ViraPower Packaging Mix was used to generate Lentivirus in 293FT cells. After infection, the stable expressing cells of miR-217 and sh-circ-TTBK2 were obtained. The lentiviruses of miR-217 were transduced in sh-circ-TTBK2 stably transfected cells to generate circ-TTBK2 (-)+miR-217 (+) cells. All experiments with nude mice were performed strictly in accordance with a protocol approved by the Administrative Panel on Laboratory Animal Care of the Shengjing Hospital. Four-week-old BALB/C athymic nude mice were purchased from the National Laboratory Animal Center (Beijing, China). The animals were free to autoclaved food and water during the experiment. The nude mice were divided into four groups: control group (only U87 or U251), circ-TTBK2 (-) group (sh-circ-TTBK2 stable expression U87 or U251 cells), miR-217 group (miR-217 stable over-expression U87 or U251 cells), and circ-TTBK2 (-)+miR-217(+) group (circ-TTBK2 inhibition and miR-217 over-expression stable U87 and U251 cells). 3 × 10^5^ cells were subcutaneously injected in the right flanks of the mice. Tumor volume was measured every 4 days when the tumors were obviously identified and the volume was calculated by the formula: volume (mm3) = length × width^2^/2. 44 days after injection, mice were sacrificed and tumors were isolated. For survival analysis in orthotopic inoculations, 3 × 10^5^ cells were stereotactically implanted into the right striatum of the mice. The number of survived nude mice was recorded, and survival analysis was performed using Kaplan–Meier survival curve.

**Reference**

1. Zhong, Z, Lv, M, and Chen, J (2016). Screening differential circular RNA expression profiles reveals the regulatory role of circTCF25-miR-103a-3p/miR-107-CDK6 pathway in bladder carcinoma. Scientific reports **6**: 30919.

2. Hansen, TB, Jensen, TI, Clausen, BH, Bramsen, JB, Finsen, B, Damgaard, CK, et al. (2013). Natural RNA circles function as efficient microRNA sponges. Nature **495**: 384-388.
